# Determinants of HIV testing intentions among heterosexual men in the Bolgatanga Municipality of Ghana: Evidence from the theory of planned behaviour

**DOI:** 10.1371/journal.pgph.0007096

**Published:** 2026-08-17

**Authors:** Dennis Bardoe, George Ahiaka, Denis Dekugmen Yar, Daniel Hayford, Robert Bagngmen Bio

**Affiliations:** 1 Department of Public Health Education, University of Skills Training and Entrepreneurial Development, Mampong, Ghana; 2 Department of Integrated Science Education, University of Skills Training and Entrepreneurial Development, Mampong, Ghana; 3 Department of Disease Control and Epidemiology, College of Health and Well-Being, Kintampo, Ghana; Islamic Azad University South Tehran Branch, IRAN, ISLAMIC REPUBLIC OF

## Abstract

Understanding intention to test for HIV reflects readiness for action and provides an opportunity for intervention before behaviour is actualised. In sub-Saharan Africa (SSA), HIV testing remains low. While numerous studies have identified factors influencing HIV testing, less is understood about how these factors shape the intention to test. Guided by the Theory of Planned Behaviour (TPB), this study examined HIV testing intention among heterosexual males in Bolgatanga Municipality. A community-based cross-sectional survey included 480 heterosexual males. Data were collected using structured questionnaires and analysed with STATA 17 and IBM SPSS AMOS 29. Statistical procedures included descriptive statistics, chi-square tests, logistic regression, confirmatory factor analysis (CFA), and structural equation modelling (SEM). Model fit was assessed using the Adjusted Goodness-of-Fit Index (AGFI), Comparative Fit Index (CFI), Goodness-of-Fit Index (GFI), Incremental Fit Index (IFI), Normed Fit Index (NFI), and Root Mean Square Error of Approximation (RMSEA). Based on a predefined mean score of ≥3.0, only 13.3% (95% CI: 10.3 - 16.4) of participants expressed a strong intention to test for HIV; 46.5% had positive attitudes, 57.7% reported high subjective norms, and 2.1% had high perceived behavioural control (PBC). Multivariate analysis revealed that positive attitudes (AOR = 1.55), high subjective norms (AOR = 2.23), and high PBC (AOR = 2.97) increased the odds of strong intention. Younger age groups had lower odds of intention, while frequent media exposure predicted higher intention. Psychometric evaluation confirmed excellent internal reliability (ω ≥ 0.96), and SEM indicated good model fit (AGFI = 0.862; CFI = 0.926; RMSEA = 0.060). PBC emerged as the strongest direct predictor (β = 0.45). HIV testing intention was influenced by psychological, social, and structural factors. Improving HIV testing among heterosexual men requires interventions that strengthen PBC, leverage supportive social norms, and expand sustained media engagement to reduce barriers and normalise HIV testing.

## Background

Human Immunodeficiency Virus (HIV) continues to pose a significant global health challenge despite remarkable biomedical advances. By the end of 2024, approximately 40.8 million people were living with HIV globally [1]. Africa continues to be the most severely affected, such that nearly one in every 25 adults lives with HIV [2]. Ghana has a relatively stable HIV prevalence rate of approximately 1.7%, despite the persistent regional disparities [3]. Across sub-Saharan Africa (SSA), heterosexual sexual contact remains the predominant mode of HIV transmission and accounts for the vast majority of new infections among adults [4]. This makes heterosexual contact the principal driver of the HIV epidemic in the region [4,5]. In Ghana, heterosexual transmission similarly accounts for most reported HIV infections [6]. Consequently, adult men remain central to both HIV transmission dynamics and prevention efforts [7]. In response to the rising cases of HIV, the World Health Organization (WHO) has emphasised the centrality of HIV testing in its “95–95–95” global targets. This strategy aims to ensure that 95% of people living with HIV know their status, 95% of those diagnosed are initiated on antiretroviral therapy (ART), and 95% of those on ART achieve viral suppression by 2030 [8]. Importantly, testing enables individuals to know their status, access timely ART, and adopt risk-reduction behaviours [9], thereby reducing morbidity, mortality, and onward transmission [8]. However, progress toward achieving the “95–95–95” global targets remains uneven, with HIV screening particularly low among men in sub-Saharan Africa, including Ghana [10].

The low testing rates among heterosexual men in this endemic region have been attributed to multiple structural, psychological, socioeconomic, and systemic barriers. Studies have documented structural barriers such as distance to facilities and cost, as well as sociocultural obstacles including stigma, gender norms, and cultural silence around HIV [11,12]. Psychological barriers, such as fear of a positive result, denial, and internalised stigma, also discourage testing [13]. While these factors are well documented in relation to actual HIV testing behaviour, less is known about how they shape intention to test. Understanding testing intention is particularly important because it reflects readiness for action and provides an opportunity for intervention before behaviour is actualised [14]. Heterosexual males represent a critical yet often underserved population within HIV prevention programmes in Ghana and across sub-Saharan Africa [7]. Although men play a central role in the dynamics of heterosexual HIV transmission, they consistently demonstrate lower engagement with HIV testing services compared with women [15]. This gap has been partly attributed to gender norms that discourage men from seeking preventive health services, limited risk perception, and sociocultural expectations surrounding masculinity [7,15]. As a result, many men remain unaware of their HIV status, which contributes to delayed diagnosis, continued transmission, and poorer treatment outcomes [16,17]. Understanding the behavioural processes that shape HIV testing intention among heterosexual males is therefore essential for designing interventions that could effectively increase testing uptake within this population [18,19].

To the best of our knowledge, no studies have systematically examined the determinants of HIV testing intention among heterosexual males in the Bolgatanga Municipality. This represents an important knowledge gap because HIV testing decisions are influenced by context-specific social, cultural, and health-system factors. The municipality is characterised by a predominantly patriarchal sociocultural environment, where gender norms, community expectations, and access to health services may shape men’s engagement with HIV testing services [20,21]. Although the municipality is served by multiple health facilities and community-based primary healthcare structures, HIV testing uptake among men in northern Ghana remains suboptimal [3,16,22]. suggesting that the availability of services alone may not be sufficient to promote testing. Examining HIV testing intention in this setting therefore provides an opportunity to identify the psychological, social, and contextual factors that influence men’s intention to test for HIV and to generate evidence that may inform interventions in similar sociocultural settings across northern Ghana and other parts of SSA.

This study sought to examine HIV testing intention among heterosexual males in the Bolgatanga Municipality using the Theory of Planned Behaviour (TPB). Specifically, the study estimated the prevalence of strong intention to test for HIV and identified the sociodemographic and TPB-related factors associated with strong HIV testing intention using logistic regression. It further examined the relationships among the latent TPB constructs and their contributions to HIV testing intention using confirmatory factor analysis (CFA) and structural equation modelling (SEM). Guided by the TPB, the study conceptualised attitude, subjective norms, and perceived behavioural control (PBC) as the primary constructs predicting intention to test for HIV. For this study, it was assumed that intention to test is higher when individuals hold favourable attitudes toward testing, perceive that others endorse HIV testing, and believe they have the ability and resources to undergo testing despite potential barriers.

## Methods

### Ethics approval and consent to participate

The study was approved by the Committee on Human Research Publications and Ethics (CHRPE), School of Medical Sciences, Kwame Nkrumah University of Science and Technology, under the Declaration of Helsinki (assigned approval number: CHRPE/AP/947/24). No personal identifiers were collected, and confidentiality was maintained through data anonymisation. The study followed harm minimisation principles consistent with the Declaration of Helsinki, with measures implemented to minimise potential psychological and social risks to participants; no adverse events were reported [23].

### Study area and justification

**Fig 1:**
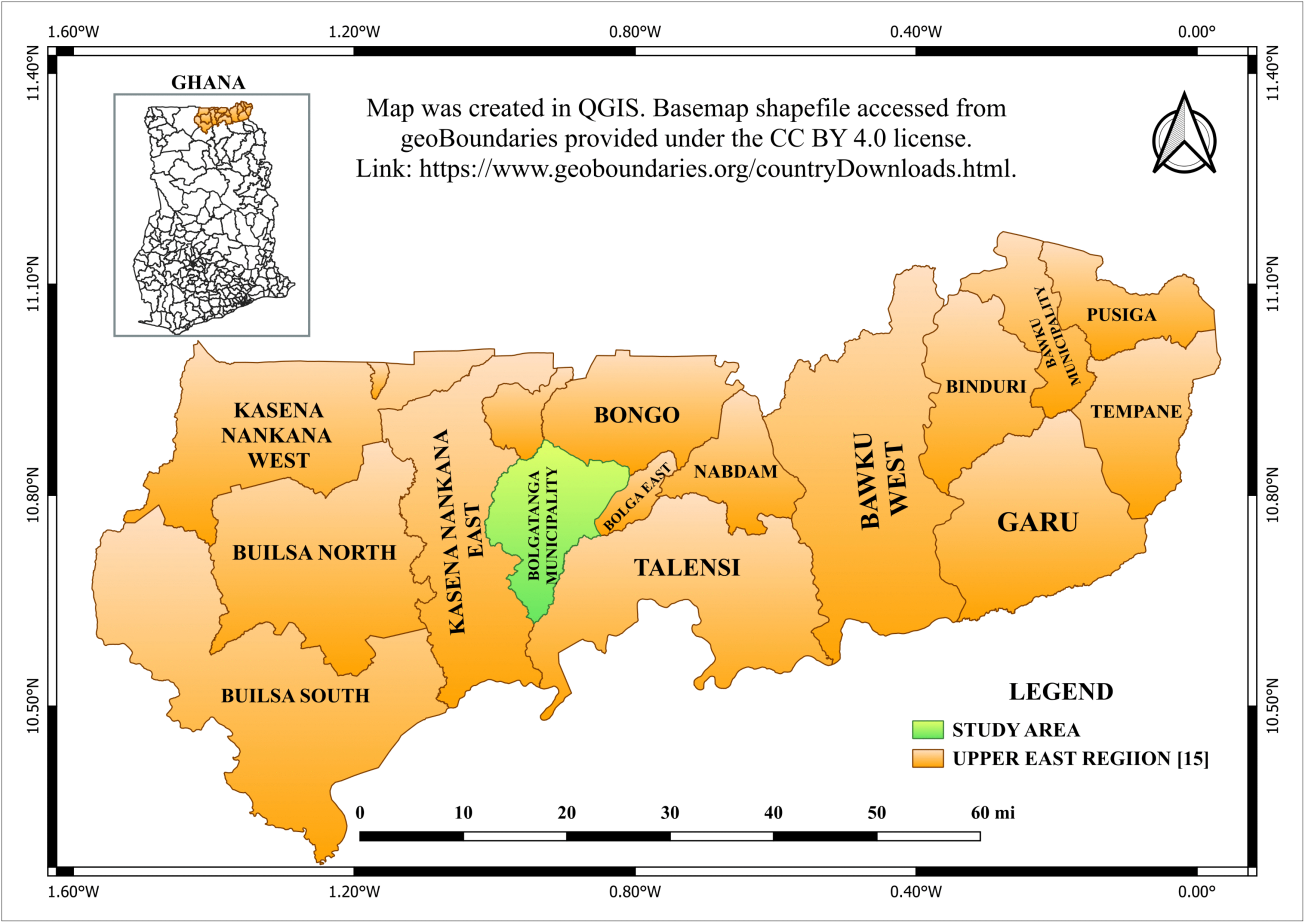
Geographic location of the Bolgatanga Municipality within Ghana. Maps were created in QGIS. Basemap shapefile accessed from geoBoundaries provided under the CC BY 4.0 license [26]. The shapefiles are publicly accessible. https://www.geoboundaries.org/countryDownloads.html.

The study was conducted in the Bolgatanga Municipality of the Upper East Region of Ghana. Fig 1 provides the geographical context of the study area within Ghana and illustrates the municipality in which the community-based sampling was undertaken. It lies in the centre of the Upper East Region, approximately between the north latitude 10°30’ and the north latitude 10°50’ and the longitudes 0°30’ and 1°00’ west of the meridian. The municipality has a total land area of 729 km^2^. It borders the Bongo District to the north, the Talensi and Nabdam Districts to the south and east, and the Kassena-Nankana District to the west. Consistent with reports from the Ghana Aids Commission in 2021, the Bolgatanga Municipality had a population of 142,509, of which 67,850 (47.6%) were males, while 74,659 (52.4%) were females [24]. In terms of health infrastructure, the municipality has 54 health facilities, consisting of three (3) hospitals, six (6) health centres, six (6) clinics, one (1) maternity home, and thirty-six (36) CHPS compounds [25]. In addition, the municipality comprises both urban and peri-urban communities with diverse socioeconomic and cultural characteristics. This provides a useful context for examining determinants of HIV testing intention within this municipality and offers insights that may inform future research in other settings with comparable sociocultural and healthcare characteristics.

### Study design and population

This study employed a community-based cross-sectional design. The design was selected because the study aimed to estimate the prevalence of HIV testing intention and simultaneously examine its associations with psychosocial constructs of the Theory of Planned Behaviour (TPB) and selected sociodemographic characteristics within a community population. Since behavioural intention and its theoretical determinants were measured concurrently rather than retrospectively, a cross-sectional design was the most appropriate approach. Moreover, this design is well suited to behavioural research that seeks to identify correlates of psychological outcomes without establishing temporal or causal relationships. The study activities were implemented sequentially, with community engagement and participant enrolment preceding the formal administration of study questionnaires.

### Theoretical foundation of the study

**Fig 2:**
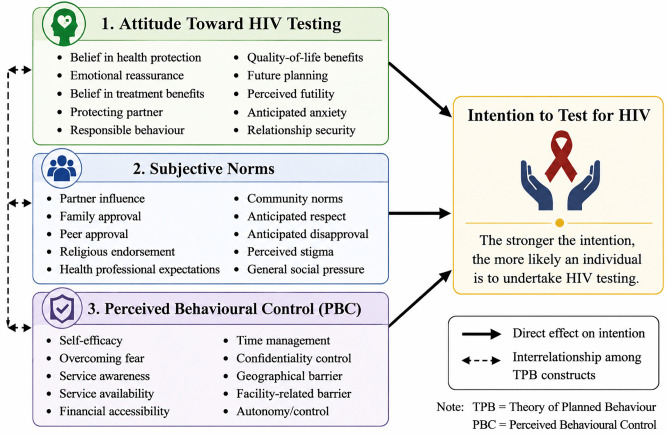
The study’s framework, informed by TPB.

This study was grounded in the TPB (Fig 2). The choice of the TPB was informed by its extensive validation across diverse health behaviours, including HIV prevention, and its ability to account for both individual cognition and contextual influences [27]. According to TPB, the most proximal determinant of a behaviour is an individual’s intention to perform it [28]. Intention, in turn, is influenced by three core constructs. These constructs include attitude toward the behaviour, subjective norms, and perceived behavioural control (PBC) [27,28]. In the context of this study, men’s likelihood of intending to test depends on their evaluation of testing (positive or negative attitudes), the perceived social pressure from family, peers, or partners (subjective norms), and the extent to which they feel capable of overcoming barriers to testing (PBC). Each construct was operationalised into multiple Likert-scale items, and composite scores were generated for statistical analysis.

### Inclusion and exclusion criteria

Eligible participants were heterosexual males, aged 18–64, permanent residents of Bolgatanga Municipality, and in good mental health, who voluntarily consented to participate. The study excluded individuals outside this age range, non-residents, those not of sound mind, or those who did not voluntarily consent. In addition, men diagnosed with HIV, including those on antiretroviral therapy (ART) or virally suppressed, were excluded, as the study specifically targeted individuals with unknown HIV status to explore predictors of HIV testing intention.

### Sample size estimation

The number of heterosexual males enrolled in the study was estimated using the Cochran formula (Equation 1).


$$\begin{array}{c} n_{\text{0}}=\frac{Z^{\text{2}}.\;p.\;(\text{1}-p)}{e^{\text{2}}} \end{array}$$ (1)

Where $n_{\text{0}}$ is the required sample size, *Z* is the standard normal deviate corresponding to a 95% confidence level (1.96), *p* is the anticipated prevalence of strong HIV testing intention, 1 – 𝑝 is the complement of prevalence (proportion of heterosexual males with weak intention to test for HIV), and *e* is the margin of error (5). Because no previous study had specifically estimated HIV testing intention among heterosexual males in the study setting, a conservative prevalence of 50% was assumed to maximise the sample size.


$$\begin{array}{c} =\frac{\left(\text{1}.\text{9}\text{6}{)}^{\text{2}}\times \text{0}.\text{5}\times (\text{1}-\text{0}.\text{5}\right)}{{\left(\text{0}.\text{0}\text{5}\right)}^{\text{2}}} \end{array}$$

$$\begin{array}{c} \approx \text{3}\text{8}\text{5} \end{array}$$

To account for the heterogeneity of the study population and the multistage sampling design, the sample size was increased by 25%, resulting in a minimum required sample of 480 participants. This adjustment was made to mitigate potential design effects, which could increase sampling variance [29]. In addition, it aimed to improve representativeness across subgroups of heterosexual males with varying sociodemographic, sociocultural, and socioeconomic characteristics. To further enhance statistical precision and ensure adequate representation across the selected communities, 500 participants were recruited.

### Sampling strategy and enrolment of participants

A multistage sampling strategy was employed to ensure representativeness of the study population. In the first stage, five communities within the Bolgatanga Municipality (Yekine, Bolga Soe, Sumbrungu, Tanzui, and Bukare) were randomly selected from the municipal list of communities. The total sample was equally distributed across these communities, as disaggregated male population data for proportional allocation were unavailable [30]. Within each community, households were chosen through systematic sampling, starting with a randomly selected household and then visiting every 5^th^ household until the target was met. Participants were selected randomly within households with multiple eligible men. Community entry, household identification, eligibility screening, and participant enrolment procedures were conducted from August 29 to September 27, 2024. Eligible participants were identified during this period, and informed consent was obtained before questionnaire administration.

### Assessment of sexual orientation

To ensure the study population consisted of heterosexual males, participants were asked about their sexual orientation and history during the eligibility screening. Specifically, they were asked whether they had ever engaged in sexual activity with another man. Given the legal and sociocultural sensitivities surrounding same-sex relations in Ghana, this information was collected confidentially and anonymously. Participants were assured that responses would remain private, with no personal identifiers linked to their answers. Neutral, non-judgmental language was used to create a safe environment for disclosure, reducing the risk of misclassification and ensuring the accuracy of the study population.

### Variables of interest

#### Outcome variable.

In this study, the outcome variable was the strong intention to test for HIV. This was assessed by asking the consenting heterosexual males to select how they agree or disagree with four items measured on a 4-point Likert scale. These items included: “*I intend to get tested for HIV within the next 3 months?*” “*I am planning to get tested for HIV the next time I have access to a testing service.*” “*I would be willing to get tested for HIV if a free and confidential service were available near me*,” and “*It is important for me to know my HIV status in the near future*”. Responses were categorically coded as “1 – Strongly Disagree, 2 – Disagree, 3 – Agree, and 4 – Strongly Agree”. To operationalise intention, the composite score derived from the four items was converted into a mean scale ranging from 1 to 4. In line with common practices in behavioural research using Likert-scale constructs, a threshold representing the upper range of agreement was used to identify individuals with a meaningful level of behavioural readiness [31–33]. A mean score of ≥ 3.0 corresponds to responses of “agree” or “strongly agree” across most intention items [34], reflecting a clear positive orientation toward undertaking HIV testing. This categorisation was therefore used to distinguish participants demonstrating a strong behavioural intention from those with weaker or uncertain intentions [31,32].

#### Main explanatory variables.

The independent variables in this study were three TPB constructs: attitudes toward HIV testing, subjective norms, and perceived behavioural control (PBC), each measured on a 4-point Likert scale. Attitudes were assessed with ten items, including two reverse-coded items for perceived futility and anticipated anxiety. Subjective norms were measured with ten items, with two reverse-coded to capture disapproval and stigma. PBC was evaluated with ten items, including two reverse-coded items for barriers like distance to facilities and lack of privacy. Composite scores were calculated by averaging item responses after reverse coding the negatively worded items, with higher scores indicating more favourable attitudes, stronger subjective norms, and greater perceived behavioural control. For descriptive analyses and logistic regression models, the composite scores for attitude, subjective norms, and PBC were dichotomised using a mean score threshold of ≥3.0. Because each item was assessed on a four-point Likert scale, a mean score of 3.0 represents the point at which responses shift from disagreement to agreement. Participants with scores ≥3.0 were therefore classified as having favourable TPB attributes, whereas those scoring <3.0 were classified as having less favourable attributes. This categorisation was used to improve interpretability of regression estimates and facilitate comparison between participants with and without favourable behavioural determinants. For confirmatory factor analysis (CFA) and structural equation modelling (SEM), however, the original item-level Likert responses were retained to preserve the latent measurement structure, maximise information, and appropriately model measurement error. Internal consistency was assessed using McDonald's omega (ω ≥ 0.70), and factor structure was verified through CFA.

### Covariates

Consistent with existing reports [35], age, marital status, educational attainment, monthly income, and media exposure were considered covariates.

### Data collection and instrumentation

Following participant enrolment and consent procedures conducted between August 29 and September 27, 2024, questionnaire-based data collection was undertaken from November 2024 to January 2025 using a structured interviewer-administered questionnaire developed based on prior research on HIV testing behaviours and the TPB constructs (S1 Text). The questionnaire covered sociodemographic characteristics, attitudes towards HIV testing, subjective norms, PBC, and intention to test. All TPB items were measured on a four-point Likert scale ranging from 1 (strongly disagree) to 4 (strongly agree). Negatively worded items were reverse-coded before composite scores were computed. Content validity was ensured through expert review and pre-testing with 10% of the sample, leading to minor revisions for clarity and cultural relevance. Internal consistency was confirmed with McDonald’s omega (ω) values above 0.80 for all domains, indicating strong reliability.

### Data management and statistical analysis

All data were entered, cleaned, and analysed using STATA 17 (StataCorp LLC, College Station, TX, USA) and IBM SPSS AMOS 29 (IBM Corp., Armonk, NY, USA). Descriptive statistics (frequencies, percentages, means, and standard deviations) summarised continuous and categorical variables. The chi-square test assessed differences across categorical variables. Logistic regression and structural equation modelling (SEM) were conducted to address complementary but distinct study objectives. Logistic regression was used to identify independent predictors of strong HIV testing intention by simultaneously examining the TPB constructs. For regression analyses, TPB construct scores were dichotomised as ≥3.00 (favourable attribute) and <3.00 (less favourable attribute), while CFA and SEM analyses used the original Likert-scale responses. Variables significant at p ≤ 0.25 were included in multivariate analysis (p < 0.05, 95% CI). Multicollinearity was checked using the Variance Inflation Factor (VIF) and tolerance statistics. A VIF value greater than 10 was considered indicative of problematic multicollinearity. In addition, a Pearson correlation matrix was generated to examine pairwise correlations among the explanatory variables included in the regression model. These procedures helped confirm that the predictors included in the final model were sufficiently independent for valid estimation.

In contrast, structural equation modelling (SEM) was performed specifically to evaluate the theoretical validity of the TPB by examining the structural relationships among the latent constructs. For confirmatory factor analysis (CFA), original Likert-scale responses were retained to preserve the ordinal structure of the indicators, item-level variance, and measurement error. Although Likert-scale responses are ordinal in nature, maximum likelihood estimation is commonly applied to multi-item Likert-type scales with four or more response categories when indicators are treated as approximately continuous, particularly when the underlying constructs are assumed to follow continuous distributions. The CFA and SEM models were therefore estimated using the maximum likelihood (ML) estimation method in IBM SPSS AMOS. ML estimation was used to obtain parameter estimates by minimising the discrepancy between the observed covariance matrix and the covariance matrix implied by the specified model. CFA evaluated item-factor loadings, with a threshold of ≥0.70 considered indicative of adequate indicator reliability [36,37]. This process ensured that only well-fitting indicators contributed to the validation of each TPB construct before structural modelling [36]. The estimation procedure generated parameter estimates, standard errors, critical ratios, standardised estimates, direct effects, indirect effects, total effects, factor score weights, and covariance estimates among model parameters. Model fit was assessed using standard indices, including the Adjusted Goodness of Fit Index (AGFI), Comparative Fit Index (CFI), Goodness of Fit Index (GFI), Incremental Fit Index (IFI), Normed Fit Index (NFI), and Root Mean Square Error of Approximation (RMSEA). Notably, sociodemographic and media-related variables were not incorporated into the SEM because they were considered external covariates rather than components of the TPB theoretical framework. Their associations with HIV testing intention were therefore evaluated separately using multivariable logistic regression, while SEM was used specifically to test the theoretical structure and pathways proposed by the TPB.

### Logistic regression model

The general model of the logistic regression equation is expressed as (Equation 2).


$$\begin{array}{c} \text{log }(p)=\;\text{ln}\left(\frac{p}{\text{1}-p}\right)=\beta_{\text{0}}+\beta_{\text{1}}X_{\text{1}}+\beta_{\text{2}}X_{\text{2}}+\ldots +\beta_{k}X_{k} \end{array}$$ (2)

Where p is the probability of having an intention to test for HIV, $\beta_{\text{0}}$ is the intercept, and $\beta_{\text{1}},\beta_{\text{2}},\ldots \beta_{k}$ are the coefficients for the explanatory variables $X_{\text{1}},\;X_{\text{2}},\ldots X_{k}$.

### Structural equation model

The general structural equation that illustrates how the exogenous latent constructs [attitude toward HIV testing, subjective norms, and PBC] influence the endogenous latent variable (intention to test for HIV) is expressed as (Equation 3):


$$\begin{array}{c} \eta =\gamma_{\text{1}}\xi_{\text{1}}+\gamma_{\text{2}}\xi_{\text{2}}+\gamma_{\text{3}}\xi_{\text{3}}+\zeta \end{array}$$ (3)

Where η is the intention to test for HIV, $\xi_{\text{1}}$ are domains for attitudes toward HIV testing, $\xi_{\text{2}}$ are subjective norms, and $\xi_{\text{3}}$ are domains for PBC, $\gamma_{\text{1}}$, $\gamma_{\text{2}}$, and $\gamma_{\text{3}}$ are structural coefficients, and ζ is the structural error term in predicting intention to test for HIV.

Secondly, the measurement model equation for the endogenous observed variable (intention to test for HIV) in the SEM is expressed as (Equation 4):


$$\begin{array}{c} {\text{y}}_{\text{1}}=\lambda_{y\text{1}}\cdot \eta +\epsilon_{\text{1}} \end{array}$$ (4)

Where ${\text{y}}_{\text{1}}$ is the observed indicator for intention to test for HIV, $\lambda_{y\text{1}}$ is the factor loading of ${\text{y}}_{\text{1}}$ on the latent variable η, η is the latent endogenous variable, and $\epsilon_{\text{1}}$ is the measurement error associated with ${\text{y}}_{\text{1}}$.

Finally, the observed items for the latent exogenous constructs [attitude toward HIV testing, subjective norms, and PBC] are defined as (Equation 5):


$$\begin{array}{c} x_{i}=\Lambda_{x}\xi +\delta \end{array}$$ (5)

Where $x_{i}$ is the vector of observed indicators for the exogenous latent construct, $\Lambda_{x}$ is the matrix of factor loadings, ξ is the vector of latent exogenous variables, and δ is the vector of measurement error in the observed exogenous indicators.

### Assessment of common method bias

Because the explanatory variables and outcome variable were collected from the same participants using the same questionnaire and at the same time point, the possibility of common method bias (CMB) was considered. To assess the extent of this potential bias, Harman’s single-factor test was conducted by entering all measurement items representing the TPB constructs and HIV testing intention into an exploratory factor analysis (EFA). The proportion of variance explained by the first unrotated factor was examined, with a value below 50% considered indicative of limited evidence of substantial common method bias.

### Sensitivity analysis using penalised logistic regression

To address the potential small-sample bias arising from the low proportion of participants, a penalised logistic regression using Firth’s correction was performed as a sensitivity analysis. Penalised likelihood estimation helps reduce small-sample bias, which may arise in conventional logistic regression models and stabilise coefficient estimates [38]. The same covariates included in the primary multivariable model were retained in the penalised regression model, and results were compared with the main analysis to assess the stability of the estimated associations.

## Results

### Sociodemographic characteristics of participants

The sociodemographic characteristics of the participants are presented in Table 1. Of the 500 male heterosexuals, 20 declined to participate. Hence, the analyses were based on data from 480 participants from five communities, namely, Yekine (20%), Bolga soe (19.6%), Sumbrungu (19.8%), Tanzui (20.4%), and Bukare (20.2%). The most common age category among participants was 28–37 years (36.7%), and more than half were not married (52.7%). Furthermore, tertiary education represented the leading educational attainment (35.8%), and Christianity was the predominant religious affiliation (48.1%). Moreover, the Frafra constituted the largest ethnic group (32.3%), and agriculture emerged as the primary occupation (31.3%). In terms of income, the highest proportion of participants reported earning between Ghȼ 100 [$9.7] and Ghȼ 500 [$45.35] per month (27.9%). Finally, media exposure was relatively high, with 41.5% reporting use almost every day.

**Table 1:** Sociodemographic characteristics of the participants.

Variable	[n]	[%]
Age [mean ± SD]	26.37 ± 3.24	[95% CI: 24.56 – 28.63]
18-27	94	19.6
28-37	176	36.7
38-47	97	20.2
48+	113	23.6
**Marital status**		
Unmarried	253	52.7
Married	164	34.2
Divorced	63	13.1
**Educational attainment**		
No formal education	17	3.5
Primary School	11	2.3
Junior High School	107	22.3
Senior High School	110	22.9
Tertiary	172	35.8
Vocational School	63	13.1
**Religious affiliation**		
Christianity	231	48.1
Islam	161	33.5
African tradition	72	15
None	16	3.3
**Ethnicity**		
Frafra	155	32.3
Kusalsi	52	10.8
Bulsa	55	11.5
Hausa	60	12.5
Mosi	70	14.6
Mamprusi	36	7.5
Others	52	10.8
**Occupational category**		
Trading	143	29.8
Agriculture	150	31.3
Transport	123	25.6
Civil service	40	8.3
Security service	24	5
**Monthly income**		
Ghȼ 100 (US$9.80) – GH¢500 (US$49.02)	134	27.9
Ghȼ 600 (US$58.82) – GH¢1,000 (US$98.04)	98	20.4
Ghȼ 1100 (US$107.84) – GH¢2,000 (US$196.08)	107	22.3
Ghȼ 2100 (US$205.88) – GH¢3,000 (US$294.12)	98	20.4
Ghȼ 3100 (US$303.92) – GH¢4,000 (US$392.16)	43	9.0
**Media exposure [Listening to the radio, watching television, and internet usage]**		
Less than once a week	120	25
At least once a week	161	33.5
Almost every day	199	41.5

n: frequency; Ghȼ: Ghana cedis; %: percentage, SD; Standard Deviation; $: US dollar. US dollar equivalents were calculated using the exchange rate on 6 June 2025 (1 US$ = Ghȼ 10.20).

### Prevalence of strong intention to test for human immunodeficiency virus (HIV)

The distribution of heterosexual males according to their responses and composite rating of intention to test for HIV is presented in Table 2. Item-level responses showed that 73.4% of participants either strongly disagreed or disagreed with the statement “I intend to get tested for HIV within the next 3 months,” whereas 26.7% agreed or strongly agreed. Similarly, 61.5% either strongly disagreed or disagreed that they were planning to get tested the next time they had access to a testing service, while 38.6% agreed or strongly agreed. Regarding willingness to test if a free and confidential service were available, 77.7% either strongly disagreed or disagreed, whereas 22.3% agreed or strongly agreed. Likewise, 71.5% either strongly disagreed or disagreed that it was important for them to know their HIV status in the near future, compared with 28.6% who agreed or strongly agreed. After composite scoring, only 13.3% (95% CI: 10.3 - 16.4) expressed a strong intention to test for HIV. The distribution across levels of intention was statistically significant (χ^2^ = 258.1, p < 0.001).

**Table 2:** Distribution of responses and composite rating of intention to test for HIV among heterosexual males.

Distribution of responses				
	Strongly Disagree	Disagree	Agree	Strongly Agree
Items	n [%]	n [%]	n [%]	n [%]
Intend to get tested for HIV within the next 3 months	117 [24.4]	235 [49.0]	98 [20.4]	30 [6.3]
Planning to get tested for HIV the next time I have access to a testing service.	97 [20.2]	198 [41.3]	154 [32.1]	31 [6.5]
Would be willing to get tested for HIV if there were a free and confidential service	100 [20.8]	273 [56.9]	93 [19.4]	14 [2.9]
It is important for me to know my HIV status in the near future.	115 [24.0]	228 [47.5]	119 [24.8]	18 [3.8]
**Composite rating of intention**				
**Categories**	**Scale**	**n [%]**	**[χ2]**	**[p]**
Strong intention	≥ 3.00	64 [13.3]	258.1	<0.001*
Weak intention	< 3.00	416 [86.7]		

n, Frequency; %, Percentage; χ2, Chi-square;

### Evaluation of the three domains of the theory of planned behaviour (TPB)

The distribution of responses across the three domains of the Theory of Planned Behaviour (TPB) is presented in Table 3. These domains included attitudes, subjective norms, and perceived behavioural control (PBC) toward HIV testing. Likewise, Table 4 provides the composite ratings of these domains and the significance of distribution across the various rating categories.

**Table 3:** Evaluation of attitude, subjective norms, and perceived behavioural control toward HIV testing.

	Distribution of responses							
	Strongly Disagree		Disagree		Agree		Strongly Agree	
Domain	[n]	[%]	[n]	[%]	[n]	[%]	[n]	[%]
**Attitudes toward HIV testing**								
Getting tested for HIV will help me protect my health.	49	10.2	75	15.6	294	61.3	62	12.9
Knowing my HIV status will give me peace of mind.	54	11.3	108	22.5	276	57.5	42	8.8
If I test positive, I can start treatment early.	47	9.8	144	30.0	243	50.6	46	9.6
HIV testing will help me protect my partner.	49	10.2	140	29.2	227	47.3	64	13.3
Testing for HIV shows responsibility.	27	5.6	32	6.7	355	74.0	66	13.8
HIV testing will improve my quality of life.	59	12.3	110	22.9	257	53.5	54	11.3
Getting tested will help me plan for the future.	54	11.3	136	28.3	232	48.3	58	12.1
HIV testing is a waste of time. (reverse coded)	56	11.7	92	19.2	254	52.9	78	16.3
HIV testing would cause unnecessary stress. (reverse coded)	50	10.4	123	25.6	238	49.6	69	14.4
Testing will make me feel safer in relationships.	42	8.8	135	28.1	242	50.4	61	12.7
**Subjective norms**								
My partner wants me to get tested.	50	10.4	98	20.4	255	53.1	77	16.0
My family supports HIV testing.	54	11.3	118	24.6	246	51.2	62	12.9
My friends think testing is important.	40	8.3	109	22.7	234	48.8	97	20.2
Religious leaders encourage testing.	48	10.0	132	27.5	187	39.0	113	23.5
Health workers expect me to test.	40	8.3	115	24.0	228	47.5	97	20.2
Most men in my community believe testing is important.	53	11.0	117	24.4	220	45.8	90	18.8
Important people would respect me if I tested.	40	8.3	107	22.3	213	44.4	120	25.0
Important people would look down on me if I tested.	50	10.4	123	25.6	176	36.7	131	27.3
My community will think badly of me if I test.	47	9.8	102	21.3	194	40.4	137	28.5
I feel pressure from others to know my status.	52	10.8	63	13.1	229	47.7	136	28.3
**Perceived behavioural control (PBC)**								
I am confident I can go for a test if I decide to.	47	9.8	115	24.0	225	46.9	93	19.4
Even if I was afraid of the results, I could still test.	141	29.4	221	46.0	106	22.1	12	2.5
I know where to go for an HIV test.	154	32.1	255	53.1	62	12.9	9	1.9
HIV testing services are available in my community.	128	26.7	260	54.2	85	17.7	7	1.5
I could afford the cost of an HIV test.	143	29.8	225	46.9	96	20.0	16	3.3
I can make time for HIV testing.	203	42.3	219	45.6	46	9.6	12	2.5
I could test without anyone knowing.	153	31.9	236	49.2	79	16.5	12	2.5
Distance would prevent me from testing. (reverse coded)	168	35.0	254	52.9	49	10.2	9	1.9
Lack of privacy in clinics would stop me. (reverse coded)	181	37.7	240	50.0	51	10.6	8	1.7
I believe I have the power to decide whether to test.	166	34.6	244	50.8	58	12.1	12	2.5

n, Frequency; %, Percentage.

**Table 4:** Composite ratings of the three domains OF TPB.

Categories	Scale	[n]	[%]	Strong intention [n] [% (95%CI)]	[χ^2^]	[p]
**Attitude towards HIV testing**						
Positive attitude	≥ 3.00	223	46.5	43 [19.3% (14.1 – 24.5)]	12.8	<0.001*
Negative attitude	< 3.00	257	53.5	21 [8.2% (4.8 – 11.5)]		
**Subjective norms**						
High subjective norms	≥ 3.00	277	57.7	51 [18.4% (13.8 – 23.0)]	14.6	<0.001*
Low subjective norms	< 3.00	203	42.3	13 [6.4% (3.0 – 9.8)]		
**Perceived behavioural control (PBC)**						
High PBC	≥ 3.00	10	2.1	5 [50.0% (12.3 – 87.7)]	11.8	<0.001*
Low PBC	< 3.00	470	97.9	59 [12.6% (9.5 – 15.6)]		

n, Frequency; %, Percentage; χ^2^, Chi-square;

#### Attitudes toward HIV testing.

For attitudes toward HIV testing (Table 3), most participants either agreed or strongly agreed that testing would help protect their health (74.2%), provide peace of mind (66.3%), demonstrate responsibility (87.8%), improve quality of life (64.8%), and support future planning (60.4%). Similarly, 60.6% agreed or strongly agreed that HIV testing would help protect their partner, while 63.1% believed that early testing would facilitate timely treatment initiation. Despite these favourable perceptions, negative views were also evident. Specifically, 69.2% agreed or strongly agreed with the reverse-coded statement that HIV testing is a waste of time, and 64.0% agreed or strongly agreed that HIV testing would cause unnecessary stress.

#### Subjective norms.

Perceptions of social support for HIV testing were mixed (Table 3). Over half of the heterosexual males agreed that their partners (53.1%), families (51.2%), and health workers (47.5%) supported HIV testing. Likewise, 48.8% agreed that friends considered testing important. Religious leaders were also viewed as supportive, with 39.0% agreeing that they encouraged testing. However, notable levels of stigma were observed, with 36.7% agreeing that people would look down on them if they tested. Similarly, 40.4% agreed that their community would think badly of them if they were tested.

#### Perceived behavioural control (PBC).

Perceived behavioural control was the weakest domain (Table 3). While 46.9% agreed that they were confident they could test if they decided to, a large proportion reported barriers. Almost half (46.0%) indicated that fear of the test result would prevent them from testing, and the majority disagreed that they knew where to obtain an HIV test (53.1%) or that testing services were available in their communities (54.2%). Similarly, 87.9% (42.3% strongly disagreed; 45.6% disagreed) reported that they could not readily make time for HIV testing. By contrast, responses to the reverse-coded privacy item indicated that 87.7% (37.7% strongly disagreed; 50.0% disagreed) did not consider lack of privacy in clinics as something that would stop them from testing. This implies that clinic privacy was perceived as less of a barrier than other structural constraints.

#### Composite ratings of TPB domains.

The distribution of participants across the three domains of the TPB is summarised in Table 4. Almost half of the respondents (46.5%) demonstrated a positive attitude toward HIV testing. In addition, a greater proportion of participants (57.7%) reported high subjective norms. Also, PBC was notably low, with only 2.1% of participants meeting the predefined criterion for high PBC based on the composite mean score (≥3.00). This skewed distribution indicates that most participants perceived substantial barriers to HIV testing. Because of the small number of participants classified as having high PBC, a sensitivity analysis using Firth's penalised logistic regression was performed to evaluate the stability of the estimated association [S1 Text**]**. The distributions across all three domains of the TPB were statistically significant (χ^2^ = 12.8, 14.6, and 11.8, *p* < 0.001, respectively).

### Predictors of HIV testing intention based on the three domains of TPB and covariates

#### Assessment of multicollinearity among predictors.

Before conducting the multivariate logistic regression analysis, multicollinearity among the independent variables was assessed using the Variance Inflation Factor (VIF) and a correlation matrix. As presented in Table 5, the pairwise correlations among the predictors were generally low to moderate. The highest correlation was observed between attitude and subjective norms (r = 0.65), while most other correlations were weak. The VIF values ranged from 1.62 to 2.33, and the tolerance values (1/VIF) ranged from 0.43 to 0.62. Since all VIF values were well below the commonly accepted threshold of 10, the results indicate that multicollinearity was not a concern among the explanatory variables included in the regression model.

**Table 5:** Correlation matrix and multicollinearity diagnostics for predictors of HIV testing intention.

Variable	Attitude	Subjective norms	PBC	Age	Marital status	Educational attainment	Monthly income	Media exposure	VIF	1/VIF
**Attitude**	1.00								2.33	0.43
**Subjective norms**	0.65	1.00							2.06	0.49
**PBC**	0.10	0.10	1.00						1.66	0.60
**Age**	0.02	0.01	0.06	1.00					1.62	0.62
**Marital status**	-0.04	-0.07	-0.01	0.61	1.00				2.22	0.45
**Educational attainment**	0.15	0.22	0.09	0.21	0.14	1.00			1.7	0.59
**Monthly income**	0.02	0.05	0.04	0.59	0.45	0.43	1.00		1.84	0.54
**Media exposure**	0.13	0.19	0.02	-0.02	-0.08	-0.02	-0.06	1.00	1.91	0.52

PBC: Perceived Behavioural Control, VIF: Variance Inflation; 1/VIF: Tolerance.

#### Predictors based on the three domains of TPB and covariates.

**Fig 3:**
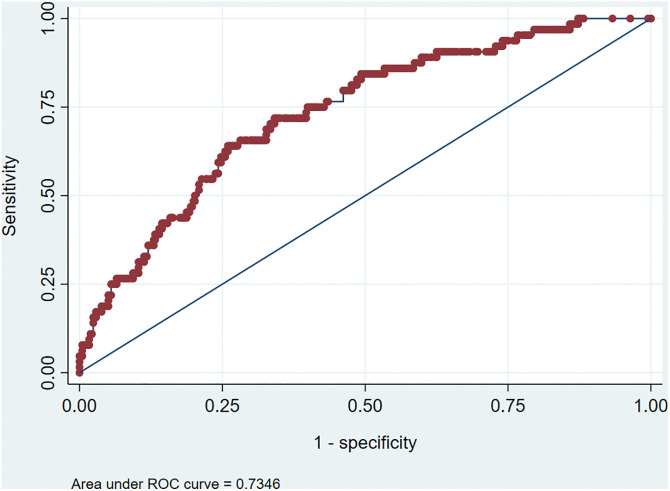
Receiver operating characteristic curve for predictors of strong HIV testing intention based on the three domains of the theory of planned behaviour and covariates.

After confirming the absence of problematic multicollinearity (Table 5), the unadjusted (**Model I**) and adjusted (**Model II**) associations between the three domains of TPB, covariates, and strong HIV testing intention were estimated using logistic regression (Table 6). The logistic regression model was statistically significant [LR χ^2^(21) = 42.85, *p* = 0.001]. Likewise, the predictors included in **Model II** approximately accounted for 51.40% of the variance in strong HIV testing intention (Pseudo R^2^ = 0.5140). In addition, the model demonstrated good discriminatory power, with an ROC AUC of 0.7346, indicating reasonable classification ability (Fig 3). The Hosmer-Lemeshow test (χ^2^(19) = 12.96, Prob > χ^2^ = 0.8405) further confirmed that the model fit the data well, with no significant discrepancy between observed and expected outcomes.

**Table 6:** Predictors of strong HIV testing intention based on the three domains of the theory of planned behaviour (TPB) and covariates.

			Model I			Model II		
Predictors	n [%]	Strong intention [n] [% (95%CI)]	COR	[95%CI]	p-value	AOR	[95%CI]	p-value
**Attitude towards HIV testing**								
Positive attitude	223 [46.5]	43 [19.3% (14.1 – 24.5)]	2.69	[1.54 - 4.68]	0.001*	1.55	[1.21 - 3.24]	0.024*
Negative attitude	257 [53.5]	21 [8.2% (4.8 – 11.5)]	1			1		
**Subjective norms**								
High subjective norms	277 [57.7]	51 [18.4% (13.8 – 23.0)]	3.29	[1.74 - 6.25]	<0.001*	2.23	[1.59 - 5.17]	0.022*
Low subjective norms	203 [42.3]	13 [6.4% (3.0 – 9.8)]	1			1		
**Perceived behavioural control (PBC)**								
High PBC	10 [2.1]	5 [50.0% (12.3 – 87.7)]	6.97	[1.95 - 9.78]	0.003*	2.97	[1.76 - 5.38]	0.017*
Low PBC	470 [97.9]	59 [12.6% (9.5 – 15.6)]	1			1		
**Age**								
18-27	94 [19.6]	10 [10.6% (4.3 – 17.0)]	0.76	[0.18 - 0.91]	0.029*	0.46	[0.18 - 0.91]	0.029*
28-37	176 [36.7]	16 [9.1% (4.8 – 13.4)]	0.54	[0.17 - 0.69]	0.003*	0.34	[0.17 - 0.69]	0.003*
38-47	97 [20.2]	22 [22.7% (14.2 – 31.2)]	0.46	[0.19 - 1.09]	0.078	0.26	[0.19 - 1.09]	0.121
48+	113 [23.6]	16 [9.1% (4.8 – 13.4)]	1			1		
**Marital status**								
Unmarried	253 [52.7]	28 [11.1% (7.2 – 15.0)]	0.68	[0.22 - 2.13]	0.213	0.86	[0.22 - 3.36]	0.229
Married	164 [34.2]	26 [15.9% (10.2 – 21.5)]	1.03	[0.32 - 3.25]	0.151	1.42	[0.24 - 3.12]	0.152
Divorced	63 [13.1]	10 [10.6% (4.3 – 16.3)]	1			1		
**Educational attainment**								
No formal education	17 [3.5]	1 [5.9% (2.3 – 7.4)]	0.24	[0.03 - 1.98]	0.186	0.41	[0.25 - 3.59]	0.215
Primary School	11 [2.3]	2 [18.2% (9.4 – 25.4)]	0.85	[0.16 - 4.45]	0.152	1.37	[0.23 - 3.08]	0.121
Junior High School	107 [22.3]	5 [4.7% (2.2 – 8.7)]	0.19	[0.06 - 1.32]	0.103	0.38	[0.11 - 1.26]	0.115
Senior High School	110 [22.9]	14 [12.7% (6.4 – 19.1)]	0.56	[0.24 - 1.28]	0.171	0.55	[0.23 - 1.32]	0.182
Tertiary	172 [35.8]	29 [16.9% (11.2 – 22.5)]	0.78	[0.37 - 1.61]	0.504	0.88	[0.39 - 1.97]	0.164
Vocational School	63 [13.1]	13 [20.6% (10.3 – 30.9)]	1			1		
**Monthly income**								
Ghȼ 100 [$9.7] and Ghȼ 500 [$45.35]	134 [27.9]	7 [5.2% (1.4 – 9.0)]	0.42	[0.13 - 1.39]	0.156	0.77	[0.17 - 3.43]	0.227
Ghȼ 600 [$54.4] and Ghȼ 1000 [$90.7]	98 [20.4]	14 [14.3% (7.2 – 21.3)]	1.27	[0.42 - 3.76]	0.171	1.59	[0.42 - 3.06]	0.199
Ghȼ 1100 [$99.8] and Ghȼ 2000 [$181.5]	107 [22.3]	21 [19.6% (12.0 – 27.3)]	1.86	[0.65 - 5.28]	0.247	2.07	[0.58 - 5.33]	0.216
Ghȼ 2100 [$190.5] and Ghȼ 3000 [$272.2]	98 [20.4]	17 [17.3% (9.7 – 25.0)]	1.59	[0.55 - 4.65]	0.192	1.9	[0.68 - 3.20]	0.218
Ghȼ 3100 [$280.8] and Ghȼ 4000 [$362.3]	43 [9.0]	5 [11.6% (6.1 – 21.6)]	1					
**Media exposure [Listening to the radio, watching television, and internet usage]**								
Almost every day	199 [41.5]	23 [12.6% (7.7 – 17.4)]	2.45	[1.34 - 3.73]	0.012*	2.12	[1.53 - 3.67]	0.011*
At least once a week	161 [33.5]	24 [14.1% (8.8 – 19.4)]	3.79	[1.85 - 5.94]	0.020*	2.79	[1.87 - 4.56]	0.016*
Less than once a week	120 [25]	17 [13.4% (7.4 – 19.4)]	1			1		

LR χ^2^ (21) = 42.85, Prob > χ^2^ = 0.001, Pseudo R^2^ = 0.514, Hosmer-Lemeshow χ^2^ (19) = 12.96, Prob > χ^2^ = 0.8405, ROC = 0.7346 [0.67 - 0.79], SE = 0.0325.

AOR: Adjusted odds ratio; COR: Crude odds ratio; HIV: Human immunodeficiency virus; *: p  <  0.05; n: frequency; Ghȼ: Ghana cedis; %: percentage; $: US dollar.

It was revealed that heterosexual males with a positive attitude toward HIV testing had significantly higher odds of expressing strong testing intention compared to those with a negative attitude (AOR = 1.55, 95% CI: 1.21 – 3.24, *p* = 0.024). In addition, participants with high subjective norms had more than twice the odds of expressing testing intention compared with those with low subjective norms (AOR = 2.23, 95% CI: 1.59 – 5.17, *p* = 0.022). Furthermore, those who reported high perceived behavioural control had nearly three times the odds of intending to test compared with those with low PBC (AOR = 2.97, 95% CI: 1.76 – 5.38, *p* = 0.017).

Moreover, heterosexual males aged 18–27 years (AOR = 0.46, 95% CI: 0.18 – 0.91, *p* = 0.029) and 28–37 years (AOR = 0.34, 95% CI: 0.17 – 0.69, *p* = 0.003) had lower odds of reporting a strong intention to test compared with those aged 48 years and above. Again, participants who reported exposure to media almost every day (AOR = 2.12, 95% CI: 1.53 – 3.67, *p* = 0.011) and at least once a week (AOR = 2.79, 95% CI: 1.87 – 4.56, *p* = 0.016) had greater odds of expressing strong intention to test compared to those who were exposed less than once a week.

#### Sensitivity analysis using penalised logistic regression.

After conducting a sensitivity analysis using penalised logistic regression, it was observed that the results were largely consistent with those obtained from the conventional logistic regression analysis. In particular, PBC remained a significant predictor of strong HIV testing intention despite the low proportion of participants reporting high PBC. The adjusted odds ratio for high PBC was 2.80 (95% CI: 1.78 – 5.97, *p* = 0.012) in the penalised model compared with 2.97 (95% CI: 1.76 – 5.38, *p* = 0.017) in the conventional model. This indicates that the observed association was not driven by sparse data bias. Similar stability in estimates was observed for positive attitude towards HIV testing, high subjective norms, age, and frequent media exposure (as shown in the supplementary information [S2 Text**]**). These findings imply that the strong association between PBC and HIV testing intention was not an artefact of sparse data arising from the low prevalence of high PBC but reflects a stable relationship within the study population.

### Assessment of common method bias

The potential for common method bias was assessed using Harman’s single-factor test. All measurement items included in the TPB constructs and HIV testing intention were entered into an exploratory factor analysis. The analysis produced multiple factors, and the first unrotated factor explained 32.0% of the total variance, which was below the conventional 50% threshold (S3 Text). This indicates that the observed relationships among attitude, subjective norms, perceived behavioural control, and HIV testing intention were unlikely to be explained solely by a common measurement method.

### Psychometric evaluation of TPB constructs predicting intention to test for HIV

Having identified both psychological and sociodemographic predictors of HIV testing intention through logistic regression, a separate structural equation modelling (SEM) was subsequently performed to evaluate the theoretical pathways proposed by the TPB. This analysis focused exclusively on the latent TPB constructs and was intended to assess the adequacy of the theoretical model rather than to estimate the effects of external covariates.

#### Measurement model and psychometric properties of TPB constructs.

**Fig 4:**
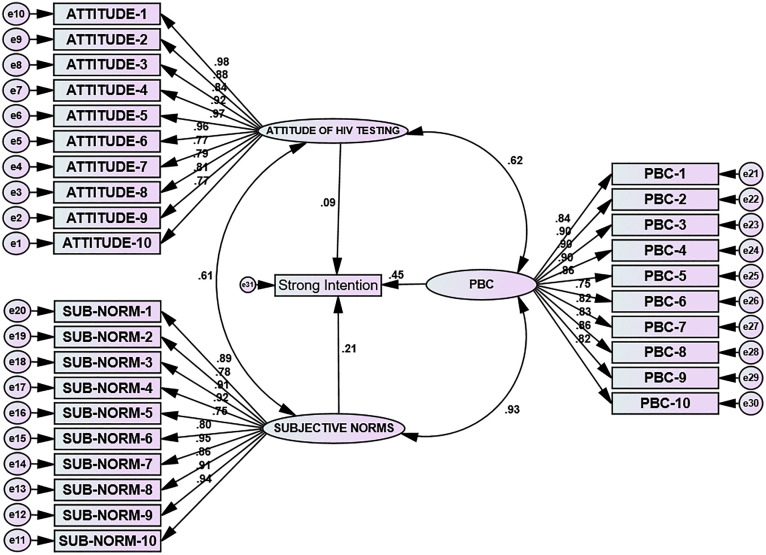
SEM model of TPB constructs predicting intention.

The measurement properties of the latent constructs underlying the TPB in predicting intention to test for HIV are presented in Table 7 and Fig 4. For the attitudes toward HIV testing construct, all 10 indicators demonstrated strong standardised factor loadings ranging from 0.768 to 0.986, with highly significant critical ratios (CR = 14.685 – 19.720, p < 0.001). The reliability of this construct was supported by a high McDonald’s omega coefficient (ω = 0.973), indicating excellent internal consistency. Indicators such as “Belief in health protection” (0.986), “Responsible behaviour” (0.970), and “Quality-of-life benefits” (0.956) were particularly strong contributors. This showed that protective and responsibility-oriented beliefs were central to positive attitudes toward HIV testing.

**Table 7:** Measurement model and psychometric properties of TPB constructs predicting intention to test for HIV.

Measuring Items	Indicator	Measurement path	Factor loading	McDonald’s omega	S. E	CR	p-value
**Latent construct: Attitudes toward HIV testing [ATTITUDE]**							
Getting tested for HIV will help me protect my health [Belief in health protection]	ATTITUDE-1	ATTITUDE-1 < --- ATTITUDE	0.986	0.973	0.05	19.720	***
Knowing my HIV status will give me peace of mind [Emotional reassurance]	ATTITUDE-2	ATTITUDE-2 < --- ATTITUDE	0.88		0.051	17.255	***
If I test positive, I can start treatment early [Belief in treatment benefits]	ATTITUDE-3	ATTITUDE-3 < --- ATTITUDE	0.836		0.054	15.481	***
HIV testing will help me protect my partner [Protecting partner]	ATTITUDE-4	ATTITUDE-4 < --- ATTITUDE	0.922		0.052	17.731	***
Testing for HIV shows responsibility [Responsible behaviour]	ATTITUDE-5	ATTITUDE-5 < --- ATTITUDE	0.97		0.05	19.400	***
HIV testing will improve my quality of life [Quality-of-life benefits]	ATTITUDE-6	ATTITUDE-6 < --- ATTITUDE	0.956		0.051	18.745	***
Getting tested will help me plan for the future [Future planning]	ATTITUDE-7	ATTITUDE-7 < --- ATTITUDE	0.768		0.052	14.769	***
HIV testing is a waste of time [Perceived futility]	ATTITUDE-8	ATTITUDE-8 < --- ATTITUDE	0.793		0.054	14.685	***
HIV testing would cause unnecessary stress [Anticipated anxiety]	ATTITUDE-9	ATTITUDE-9 < --- ATTITUDE	0.808		0.052	15.538	***
Testing will make me feel safer in relationships [Relationship security]	ATTITUDE-10	ATTITUDE-10 < --- ATTITUDE	0.768		0.05	15.360	***
**Latent construct: Subjective norms [SUB-NORM]**							
My partner wants me to get tested [Partner influence]	SUB-NORM-1	SUB-NORM-1 < --- SUB-NORM	0.886	0.972	0.026	34.077	***
My family supports HIV testing [Family approval]	SUB-NORM-2	SUB-NORM-2 < --- SUB-NORM	0.781		0.032	24.406	***
My friends think testing is important [Peer approval]	SUB-NORM-3	SUB-NORM-3 < --- SUB-NORM	0.911		0.025	36.440	***
Religious leaders encourage testing [Religious endorsement]	SUB-NORM-4	SUB-NORM-4 < --- SUB-NORM	0.924		0.026	35.538	***
Health workers expect me to test [Health professional expectations]	SUB-NORM-5	SUB-NORM-5 < --- SUB-NORM	0.751		0.032	23.469	***
Most men in my community believe testing is important [Community norms]	SUB-NORM-6	SUB-NORM-6 < --- SUB-NORM	0.805		0.029	27.759	***
Important people would respect me if I tested [Anticipated respect]	SUB-NORM-7	SUB-NORM-7 < --- SUB-NORM	0.947		0.022	43.045	***
Important people would look down on me if I tested [Anticipated disapproval]	SUB-NORM-8	SUB-NORM-8 < --- SUB-NORM	0.861		0.03	28.700	***
My community will think badly of me if I test [Perceived stigma]	SUB-NORM-9	SUB-NORM-9 < --- SUB-NORM	0.91		0.025	36.400	***
I feel pressure from others to know my status [General social pressure]	SUB-NORM-10	SUB-NORM-10 < --- SUB-NORM	0.944		0.029	32.552	***
**Latent construct: Perceived behavioural control [PBC]**							
I am confident I can go for a test if I decide to [Self-efficacy]	PBC-1	PBC-1 < --- PBC	0.841	0.961	0.041	20.512	***
Even if afraid of the results, I could still test [Overcoming fear]	PBC-2	PBC-2 < --- PBC	0.904		0.042	21.524	***
I know where to go for an HIV test [Service awareness]	PBC-3	PBC-3 < --- PBC	0.896		0.04	22.400	***
HIV testing services are available in my community [Service availability]	PBC-4	PBC-4 < --- PBC	0.897		0.043	20.860	***
I could afford the cost of an HIV test [Financial accessibility]	PBC-5	PBC-5 < --- PBC	0.863		0.04	21.575	***
I can make time for HIV testing [Time management]	PBC-6	PBC-6 < --- PBC	0.749		0.045	16.644	***
I could test without anyone knowing [Confidentiality control]	PBC-7	PBC-7 < --- PBC	0.823		0.041	20.073	***
Distance would prevent me from testing [Geographical barrier]	PBC-8	PBC-8 < --- PBC	0.833		0.042	19.833	***
Lack of privacy in clinics would stop me [Facility-related barrier]	PBC-9	PBC-9 < --- PBC	0.86		0.04	21.500	***
I believe I have the power to decide whether to test [Autonomy/control]	PBC-10	PBC-10 < --- PBC	0.82		0.043	19.070	***

C.R: Critical Ratio; S.E: Standard Error; ***: p < 0.001.

For the subjective norms construct, the factor loadings ranged from 0.751 to 0.947. The critical ratios were also statistically significant (CR = 23.469 – 43.045, p < 0.001). The construct achieved very high internal reliability (ω = 0.972). Among the strongest indicators were “Anticipated respect” (0.947), “General social pressure” (0.944), and “Religious endorsement” (0.924), suggesting that perceived respect and community/religious expectations play a critical role in shaping normative pressure toward HIV testing.

Finally, the PBC construct was also significant, with factor loadings ranging from 0.749 to 0.904 (CR = 16.644 – 22.400, p < 0.001) and excellent reliability (ω = 0.961). Strong indicators included “Overcoming fear” (0.904), “Service availability” (0.897), and “Service awareness” (0.896), reflecting the importance of psychological readiness and structural access in enabling HIV testing behaviour.

#### Predictive strength of the TPB model in explaining variance in intention to test for HIV.

The structural equation model (SEM) illustrated in Fig 4 depicts the predictive pathways of TPB constructs in explaining heterosexual males’ strong intention to test for HIV. Overall, the model (Fig 4) supports the predictive validity of the TPB model in this context. Thus, it highlights that attitudes, subjective norms, and PBC are strongly interlinked and jointly explain variation in the intention to undergo HIV testing among heterosexual males. As shown in Table 8, all model fit indices [AGFI (0.862), CFI (0.926), GFI (0.913), IFI (0.924), NFI (0.918), and RMSEA (0.060)] met or exceeded their respective acceptable thresholds. Further, covariances and correlations among the latent constructs were statistically significant (p < 0.001). The strongest correlation was observed between subjective norms and PBC (r = 0.93), followed by attitude and PBC (r = 0.622), and attitude and subjective norms (r = 0.611). These imply that subjective norms exert a particularly strong influence on PBC, while attitudes and subjective norms are also highly interrelated. Moreover, the model shows that PBC emerged as the strongest direct predictor of strong intention (β = 0.45), followed by subjective norms (β = 0.21). In contrast, attitude toward HIV testing had only a weak direct effect on intention (β = 0.09). These observations stress that favourable attitudes alone may not be sufficient to translate into strong behavioural intentions without social support and perceived control.

**Table 8:** Predictive strength of the TPB model in explaining variance in intention to test for HIV.

Model fit indices					
AGFI	CFI	GFI	IFI	NFI	RMSEA
0.862	0.926	0.913	0.924	0.918	0.060
**Acceptable threshold for model fit indices**					
> 0.80	≥ 0.90	> 0.90	> 0.90	> 0.90	< 0.08
**Remarks after comparison with acceptable thresholds**					
Excellent fit	Excellent fit	Excellent fit		Excellent fit	Excellent fit
**Covariances and correlations among latent constructs influencing intention to test for HIV**					
	**Covariances**	**S.E.**	**C.R.**	**Correlation**	**P**
ATTITUDE < -- > SUB-NORM	1.205	0.118	10.212	0.611	***
SUB-NORM < -- > PBC	2.201	0.166	13.259	0.93	***
ATTITUDE < -- > PBC	1.078	0.108	9.981	0.622	***

AGFI: Adjusted Goodness of Fit Index; CFI: Comparative Fit Index; C.R: Critical Ratio; GFI: Goodness of Fit Index; IFI: Incremental Fit Index; NFI: Normed Fit Index; RMSEA: Root Mean Square Error of approximation; S.E: Standard Error; ***: p < 0.001.

## Discussion

### Prevalence of strong intention to test for human immunodeficiency virus (HIV)

This study assessed the intention to test for HIV among heterosexual males in the Bolgatanga Municipality. It revealed that only 13.3% (95% CI: 10.3 – 16.4) of the participants expressed a strong intention to undergo HIV testing. This aligns with previous studies, which have similarly documented low levels of intention or willingness to test for HIV [39]. The convergence of these findings implied that despite ongoing global efforts to normalise HIV testing as an entry point to prevention and treatment, significant gaps persist in male engagement [18]. In many high-income settings, community-based strategies, workplace testing programs, and partner-centred interventions have helped improve male testing rates [18,40]. By contrast, the persistently low intention observed in this study indicates that such interventions may be either limited in scope, poorly adapted to local realities, or inadequately accessible to men. Several factors may explain the low intention to test for HIV among heterosexual males. These include structural barriers such as inadequate male-friendly testing services, limited community outreach, long waiting times, and stigma at testing centres [41]. Also, sociocultural barriers, including prevailing gender norms which often frame health-seeking as a “female responsibility,” and the perception that testing is unnecessary unless symptomatic, may play a role in the low intention observed [42]. Moreover, misconceptions about HIV, fear of positive results, and concerns about confidentiality also remain powerful deterrents [43]. Finally, psychological barriers, including denial of risk and low perceived susceptibility, may further undermine intention, particularly among men who consider themselves healthy or monogamous [44]. In general, the low level of strong intention to test for HIV observed in this study not only reflects the broader challenge of engaging men in HIV prevention but also highlights the need for innovative, gender-sensitive approaches. This stresses the need to integrate HIV testing into routine health services, communication strategies that normalise male testing, strengthen confidentiality, and address stigma through community sensitisation. Ultimately, these are likely to reinforce the ambitious targets of universal HIV testing and early treatment initiation.

### Evaluation of the three domains of the theory of planned behaviour (TPB)

This study also examined the distribution of attitudes, subjective norms, and perceived behavioural control (PBC) toward HIV testing among heterosexual males. It revealed a relatively high prevalence of positive attitudes toward HIV testing and high subjective norms. By contrast, PBC was low. The relatively high prevalence of positive attitudes towards HIV testing aligns with findings from similar studies, where awareness of the benefits of early diagnosis and treatment has improved over time [45–47]. This indicates that public health messaging and HIV prevention campaigns may have successfully influenced perceptions, fostering acceptance of HIV testing as an important health behaviour [45]. Moreover, the predominance of high subjective norms also suggests that social influences such as encouragement from peers, family, or partners are crucial in motivating men toward testing. Previous research has shown that men often rely on perceived community expectations and partner support when deciding whether to test for HIV [48,49]. This finding highlights the potential value of leveraging social networks and community leaders as key players to reinforce positive behavioural intentions.

However, the low level of PBC echoes a critical gap despite positive attitudes and strong normative pressures. Most participants reported limited control over practical aspects of HIV testing, including fear of receiving a positive result, limited awareness of where services were available, inadequate local service availability, and difficulty making time for testing. Interestingly, responses to the reverse-coded privacy item implied that lack of privacy in clinics was not widely perceived as a barrier. This indicates that concerns about confidentiality within health facilities may have been less influential than other structural and psychological constraints. These findings mirror earlier studies conducted in low-resource settings, where fear of HIV diagnosis, service accessibility, and logistical challenges often reduce men's perceived ability to seek testing [43,50]. This low PBC may weaken the translation of favourable attitudes and social expectations into actual testing behaviours. This finding shows that interventions aimed at improving HIV testing may benefit from going beyond messaging and normative reinforcement to focus on reducing structural and psychological barriers. Likewise, this finding also connotes that interventions should prioritise reducing practical barriers, improving awareness of available HIV testing services, and helping men overcome fear of receiving a positive diagnosis, rather than focusing solely on concerns about privacy within clinics. Therefore, expanding male-friendly and confidential testing services, integrating testing into routine health checks, and addressing stigma through education could enhance men’s sense of control.

### Predictors of HIV testing intention

This study revealed several important predictors of strong HIV testing intention. Notably, while the direction of associations observed in this study is consistent with earlier research, the relative strength of the predictors differs in important ways. It revealed that heterosexual males with a positive attitude toward HIV testing had significantly higher odds of expressing a strong intention to undergo HIV testing. This aligns with the TPB proposition that favourable evaluations of a behaviour enhance the likelihood of building an intention [27,28]. Similar findings have been reported across sub-Saharan Africa, where men who view HIV testing as beneficial for protecting their health and that of their partners are more likely to express willingness to test [16,19]. In many communities, HIV testing is often associated with fear of judgment or being labelled as promiscuous [51]. Heterosexual males who perceive HIV testing as beneficial either for safeguarding their own health or protecting their partners may feel reassured that the benefits outweigh perceived risks such as stigma or discrimination [52]. Therefore, a positive attitude may reflect greater trust in the healthcare system, prior exposure to health campaigns, or personal experiences that normalised testing [53]. These observations indicate that health education campaigns that foster positive attitudes and challenge misconceptions surrounding HIV testing may be useful for promoting HIV testing intention.

In addition, heterosexual males with high subjective norms had more than twice the odds of expressing stronger testing intention. This highlights the influence of social expectations, peer dynamics, and interpersonal support in shaping men’s health decisions. Previous studies have demonstrated that endorsement from partners, friends, and community leaders plays a decisive role in encouraging men to test for HIV [48,49]. In the Ghanaian context, decision-making among men is often influenced by peer groups, family members, and partners [54]. When social networks endorse HIV testing as a responsible and protective act, individuals may feel encouraged to conform to such expectations. Conversely, in settings where testing is associated with shame or suspicion, social pressure may discourage participation [50]. Thus, the significant role of subjective norms suggests that interpersonal and community-level influences are powerful drivers of male engagement with HIV prevention. Therefore, interventions such as peer-led programs, partner-involvement strategies, and community-driven advocacy may be particularly effective in strengthening testing intentions.

PBC was also a significant predictor, with participants reporting high PBC being nearly three times more likely to intend to test. Although only a small proportion of participants (2.1%) met the criterion for high PBC, this does not necessarily contradict its strong association with HIV testing intention. The low prevalence primarily reflects the stringent operational definition of high PBC, which required consistently positive responses across multiple indicators of self-efficacy, autonomy, and perceived access to testing services. Participants who satisfied this criterion appeared to possess substantially greater confidence and perceived ability to overcome barriers to HIV testing than the rest of the sample. Nevertheless, the consistency of the findings in the sensitivity analysis using Firth’s penalised logistic regression suggests that the observed association was unlikely to be an artefact of sparse data bias. Instead, the findings indicate that although relatively few men perceived themselves as having sufficient control over HIV testing, those who did were substantially more likely to express a strong intention to test. This echoes the critical role of agency and confidence in overcoming barriers to HIV testing. Prior research indicates that challenges such as stigma, fear of confidentiality breaches, financial limitations, and poor access to services undermine men’s sense of control, thereby weakening their intentions [50,55]. On the contrary, heterosexual males who perceive themselves as having the resources, knowledge, and autonomy to overcome these barriers are therefore more likely to express testing intention. Hence, interventions that address these barriers through confidential, male-friendly, and accessible services could empower men with greater control and consequently enhance their likelihood of testing.

Age was another significant determinant, with younger men (18–27 and 28–37 years) showing lower odds of strong testing intention. This finding is consistent with previous reports that have strongly maintained that younger males are less likely to test for HIV [17,56]. While some evidence suggests that younger adults may test more due to greater exposure to health campaigns [57], the reduced intention observed in this study may be attributed to stigma, denial of risk, or limited autonomy in health-seeking behaviour [50]. Younger men may also underestimate their susceptibility to HIV, thereby deprioritising testing [58]. These dynamics highlight the need for youth-focused interventions that emphasise risk perception, confidentiality, and accessibility of services personalised to younger populations. While the present study did not formally examine whether the relationship between age and testing intention was mediated by TPB constructs, the findings could be interpreted within the TPB framework. The lower testing intention observed among younger men may be associated with weaker PBC arising from concerns about confidentiality, limited familiarity with available testing services, or reduced confidence in overcoming anticipated barriers [27]. It may also indicate weaker subjective normative influences if younger men perceive less encouragement from peers, partners, or other important referents to undergo HIV testing [28]. These interpretations are consistent with the TPB, which proposes that behavioural intention is strengthened when individuals perceive supportive social norms and believe they have the capability and opportunity to perform the behaviour [27,28]. Future studies should explicitly examine whether age differences in HIV testing intention are mediated by perceived behavioural control, subjective norms, or both.

Finally, frequent media exposure was positively associated with strong HIV testing intention. As such, heterosexual males exposed to radio, television, or internet messaging almost daily or at least weekly were significantly more likely to express a strong intention. Consistent with previous research, sustained exposure to media campaigns motivates individuals to engage in preventive health practices [51,59]. The observed association may be attributed to repeated reinforcement of HIV prevention messages, normalisation of testing behaviours, and debunking of myths about HIV transmission and treatment [60]. In Ghana, where the media is a primary channel for public health communication, consistent exposure may shift perceptions from fear and stigma toward empowerment and responsibility [61]. Limited exposure, by contrast, may perpetuate misinformation and low risk perception. Strengthening media-driven interventions with consistent, culturally relevant, and relatable messaging could further enhance testing intentions among heterosexual males.

### Psychometric evaluation of TPB constructs predicting intention to test for HIV

The psychometric properties and predictive validity of TPB constructs in explaining heterosexual males’ intention to test for HIV were also assessed. The confirmatory factor analysis (CFA) confirmed the structural validity and internal reliability of the three latent constructs. All constructs demonstrated consistently high factor loadings and excellent internal consistency, highlighting that the indicators were both theoretically sound and empirically fit. This reinforces the multidimensional nature of HIV testing intentions, in which personal beliefs, social influences, and perceived control over testing decisions interact to shape behavioural readiness [62,63].

The strong contributions of specific indicators within each construct offer insights into the contextual drivers of HIV testing intention. For instance, within the attitude domain, beliefs related to health protection, responsibility, and quality-of-life benefits emerged as the most influential. This shows that HIV testing is perceived not only as a safeguard to personal health but also as a moral and social responsibility, aligning with broader cultural expectations of responsible masculinity [64]. Similarly, the dominance of respect, social pressure, and religious endorsement among the subjective norms indicators underlines the powerful role of community and faith-based influences in guiding male health behaviour in Ghana [54]. For PBC, overcoming fear, service availability, and service awareness highlight both psychological readiness and structural access as pivotal enablers of testing behaviour.

The structural model further confirmed the predictive strength of the TPB framework in this population, with acceptable to excellent model fit indices across all parameters. Significant covariances between constructs highlight that HIV testing intention is rarely determined by a single domain but rather emerges from dynamic interrelationships between social expectations, individual attitudes, and structural enablers [50]. The particularly strong correlation between subjective norms and PBC indicates that normative pressures may enhance or constrain individuals’ perceptions of control. In practical terms, when men perceive community endorsement of HIV testing, they may also feel more empowered to access services, thus reinforcing their behavioural control [19]. The observed high intercorrelations among TPB constructs, particularly between subjective norms and PBC, underline the socially embedded nature of HIV testing decisions in this setting. While attitudes represent personal evaluations of testing, subjective norms reflect perceived social endorsement, and PBC captures perceived agency and access. These domains may converge in environments where social approval directly facilitates or constrains behavioural autonomy. In such contexts, men who perceive strong normative support from partners, family members, or community leaders may simultaneously feel more empowered and capable of accessing testing services. This convergence is consistent with prior TPB-based research in collectivist and resource-constrained settings, where social influence and structural control are tightly intertwined rather than operating as isolated determinants [65,66].

Importantly, the structural path analysis revealed that PBC had the strongest direct effect on intention, followed by subjective norms, whereas attitude exerted only a weak direct influence. The prominence of PBC as the strongest predictor distinguishes this study from several earlier investigations in which PBC played a secondary role [67,68]. This difference may be attributable to contextual realities in northern Ghana, where structural barriers such as limited-service availability, concerns about privacy, and fear of social exposure remain pronounced [69]. In such settings, intention appears to be less about whether men value HIV testing and more about whether they believe testing is realistically achievable without social or personal cost. This finding implies that while favourable beliefs toward HIV testing are necessary, they may not be sufficient to translate into strong behavioural intentions unless supported by enabling environments and social approval [45]. In contexts where stigma, fear, and structural barriers remain significant, positive attitudes alone may fail to trigger testing behaviour. Instead, social reinforcement from peers, partners, and religious leaders, combined with men’s confidence in accessing services, appears to be the key driver of intention [70]. This finding reinforces the argument that HIV testing interventions for men must move beyond informational strategies toward structural and service-delivery reforms that actively reduce perceived and real constraints.

### Implications for policy and practice

Several actionable strategies could be drawn from these findings. Firstly, community mobilisation and peer-led campaigns may be leveraged to normalise HIV testing, especially by emphasising responsibility, respect, and protection of partners. In addition, engaging religious and traditional leaders to endorse testing could further strengthen subjective norms, as these figures hold substantial influence over men’s health decisions. Furthermore, efforts to enhance PBC should prioritise male-friendly, accessible, and confidential testing services. These may address barriers such as stigma, lack of privacy, and inconvenient service hours. Similarly, outreach models, including mobile clinics and workplace-based testing, could reduce practical constraints related to time and distance. Moreover, mass media campaigns should continue to highlight the protective and quality-of-life benefits of HIV testing while dispelling myths and fears. Such campaigns could be particularly effective when sustained and targeted at younger males, who may have lower perceived vulnerability. Finally, integrating HIV testing promotion into broader men’s health initiatives could increase uptake by embedding it within trusted health platforms. By combining strategies that strengthen individual motivation, enhance normative support, and remove structural barriers, policymakers and program implementers could create enabling environments that convert intention into actual HIV testing behaviour.

### Study’s strengths and limitations

Unlike many previous studies, this study applied the TPB as a comprehensive theoretical framework to capture the interplay between attitudes, subjective norms, and PBC in shaping behavioural intentions. This multidimensional approach provided a deeper understanding of the psychological, social, and structural drivers of HIV testing intention. Also, the use of CFA and SEM enabled rigorous testing of the validity, reliability, and interrelationships among the TPB constructs, while also accounting for measurement error. Although only a small proportion of participants reported high perceived behavioural control, the sensitivity analysis using penalised logistic regression produced estimates similar to those of the main model. This consistency suggests that the observed association between PBC and HIV testing intention was unlikely to be an artefact of sparse data bias.

Despite its strengths, the study has some limitations. The cross-sectional design limits causal inference, hindering the establishment of temporal relationships between TPB constructs and HIV testing intention. Although efforts were made to ensure confidentiality, reliance on self-reports, particularly regarding HIV testing and sexual orientation, may have led to underreporting or misclassification due to the sociocultural stigma surrounding HIV and same-sex relationships in Ghana. In addition, the high correlations observed between some TPB constructs, especially subjective norms and PBC, suggest potential conceptual overlap, possibly influenced by shared sociocultural factors. While these constructs are distinct in theory, their strong empirical link may reflect simultaneous social approval and stigma pressures. Nonetheless, the validated theoretical framework and strong psychometric performance of the TPB constructs bolster the transferability of these findings to similar Ghanaian contexts. Even though the TPB composite scores were dichotomised for logistic regression to facilitate interpretation, this approach may have reduced variability and statistical power compared with modelling the constructs as continuous predictors. Nevertheless, the primary psychometric and structural analyses retained the original item-level responses, thereby preserving the continuous latent structure of the TPB constructs. Furthermore, because all variables were collected using a single questionnaire administered at one time point, the possibility of common method bias cannot be completely excluded. Although Harman’s single-factor test did not indicate substantial common method bias, and several methodological measures were implemented to minimise this risk, including participant confidentiality, use of theoretically grounded constructs, reverse-coded items, and validation through confirmatory factor analysis, residual measurement bias may still exist. Future studies could strengthen causal interpretation and reduce common method concerns by incorporating longitudinal designs, objective measures of HIV testing behaviour, or data collected from multiple sources.

## Conclusion

This study revealed that only a small proportion of heterosexual males expressed a strong intention to test for HIV. Beyond this headline finding, the study demonstrated that intention was shaped by a constellation of psychological, social, and structural determinants within the framework of the Theory of Planned Behaviour (TPB). Positive attitudes, strong subjective norms, and high perceived behavioural control (PBC) significantly increased the odds of expressing stronger testing intention. Sociodemographic factors, particularly age and media access, also influenced testing intention. Therefore, efforts to improve HIV testing uptake among heterosexual males should move beyond single-dimensional interventions to integrated, multilevel strategies. Interventions should prioritise enhancing self-efficacy and autonomy in decision-making, strengthening community and peer support to shift normative expectations, and improving service accessibility through awareness, availability, and male-friendly testing environments. In addition, sustained media campaigns that normalise HIV testing could further reinforce positive attitudes and normative support. By simultaneously addressing attitudinal, normative, and control-related barriers, Ghana could substantially increase HIV testing uptake among men, thereby contributing to national and global targets for HIV prevention and control.

## Supporting information

S1 Data Dataset. (XLSX)

S1 Text Questionnaire. (DOCX)

S2 Text Sensitivity analysis. (DOCX)

S3 Text Harman’s single-factor test for assessment of common method bias. (DOCX)
