# Supplementary material for: Determinants of HIV testing intentions among heterosexual men in the Bolgatanga Municipality of Ghana: Evidence from the theory of planned behaviour
Source: PLOS Glob Public Health. 2026 Aug 17;6(8):e0007096. doi: 10.1371/journal.pgph.0007096 (PMC13480663; doi:10.1371/journal.pgph.0007096)
Supplement: Supplementary file 1 [file pgph.0007096.s002.docx]

BARRIERS TO HIV TESTING AMONG HETEROSEXUAL MALES IN BOLGATANGA MUNICIPALITY

**INTRODUCTION**

This questionnaire is part of a research study aimed at understanding the social, economic, and psychological factors that influence HIV testing among heterosexual men in the Bolgatanga Municipality of Ghana. Your responses will help identify the key barriers preventing men from getting tested for HIV and contribute to designing better health interventions. Your participation is voluntary, and all information you provide will be treated as confidential. The survey will take approximately 10 – 15 minutes to complete. No personal identifiers will be collected. By continuing, you consent to take part in this study.

# SECTION A: DEMOGRAPHIC CHARACTERISTICS

This section collects background information such as your age, marital status, education, employment, and residence. This will help us understand how personal and social factors relate to HIV testing behaviour.

Age………………………………………………………………………………….

| I would like to start by asking you a few questions about yourself. | | | |
| --- | --- | --- | --- |
| No. | Question | Response options | Skip |
| 1. | Age range | 1. Between 18 and 27 [ ] 2. Between 28 and 37 [ ] 3. Between 38 and 47 [ ] 4. Between 48 and 57 [ ] 5. Between 58 and 64 [ ] |  |
| 2. | Marital Status: | 1. Unmarried [ ] 2. Divorced [ ] 3. Married [ ] |  |
| 3. | Education attainment | 1. No formal education [ ] 2. Primary [ ] 3. JHS/Middle School [ ] 4. SHS [ ] 5. Technical Vocational Education [ ] 6. Tertiary [ ] |  |
| 4. | Religious Affiliation | 1. Christianity [ ] 2. Islam [ ] 3. African Traditional Religion [ ] |  |
| 5. | Ethnicity | 1. Frafra [ ] 2. Kusalsi [ ] 3. Bulsa [ ] 4. Hausa [ ] 5. Mosi [ ] 6. Mamprusi [ ] |  |
| 6. | Occupational category | 1. Trading [ ] 2. Agriculture [ ] 3. Transport [ ] 4. Civil service [ ] 5. Security service [ ] |  |
| 7. | On the average, how much do you earn from your work monthly? | 1. Between Ghȼ 100 and Ghȼ 500 [ ] 2. Between Ghȼ 600 and Ghȼ 1000 [ ] 3. Between Ghȼ 1100 and Ghȼ 2000 [ ] 4. Between Ghȼ 2100 and Ghȼ 3000 [ ] 5. Between Ghȼ 3100 and above [ ] |  |
| 8. | Media exposure (Listening to radio, watching television, and internet usage) | 1. Less than once a week [ ] 2. At least once a week [ ] 3. Almost every day [ ] |  |
| 9. | Frequency of visits to health facility last 3 months | 1. None [ ] 2. Once [ ] 3. Twice or more [ ] |  |
| 10. | Place of residence | 1. Rural [ ] 2. Urban [ ] |  |
| 11. | National health insurance coverage | 1. No [ ] 2. Yes [ ] |  |
| 12. | Experienced any reproductive tract infection in the past 6 months | 1. No [ ] 2. Yes [ ] |  |

# SECTION B: HIV TESTING BEHAVIOUR

This section asks about your past experience with HIV testing, whether you have tested before, how recently, and how often. This information is central to our study.

| No. | Question | Response options | Skip |
| --- | --- | --- | --- |
| 13. | Have you ever tested for HIV? | 1. Yes [ ] 2. No [ ] |  |
| 14. | If yes, when was your last HIV test? | 1. Within last 3 months [ ] 2. 3 – 6 months ago [ ] 3. 6 – 12 months ago [ ] 4. More than a year ago [ ] 5. I have not tested [ ] |  |
| 15. | How many times have you tested for HIV? | 1. Once [ ] 2. 2–3 times [ ] 3. More than 3 times [ ] 4. I have not tested [ ] |  |

# SECTION C: INTENTION TO TEST FOR HIV

This section explores your willingness or intention to test for HIV in the future. It helps us understand motivational factors and potential readiness to take up testing services.

| 16. | I intend to get tested for HIV within the next 3 months. | 1. Strongly disagree [ ] 2. Disagree [ ] 3. Agree [ ] 4. Strongly agree [ ] |  |
| --- | --- | --- | --- |
| 17. | I am planning to get tested for HIV the next time I have access to a testing service. | 1. Strongly disagree [ ] 2. Disagree [ ] 3. Agree [ ] 4. Strongly agree [ ] |  |
| 18. | I would be willing to get tested for HIV if a free and confidential service were available near me. | 1. Strongly disagree [ ] 2. Disagree [ ] 3. Agree [ ] 4. Strongly agree [ ] |  |
| 19. | It is important for me to know my HIV status in the near future. | 1. Strongly disagree [ ] 2. Disagree [ ] 3. Agree [ ] 4. Strongly agree [ ] |  |

# SECTION D: SOCIOCULTURAL BARRIERS

This section looks at how cultural expectations, social stigma, and community beliefs may influence your decision to undergo HIV testing.

| 20. | Talking about HIV is considered a taboo in my community. | 1. Strongly disagree [ ] 2. Disagree [ ] 3. Agree [ ] 4. Strongly agree [ ] |  |
| --- | --- | --- | --- |
| 21. | I would be ashamed if people found out I tested for HIV. | 1. Strongly disagree [ ] 2. Disagree [ ] 3. Agree [ ] 4. Strongly agree [ ] |  |
| 22. | My religious beliefs discourage me from getting tested for HIV. | 1. Strongly disagree [ ] 2. Disagree [ ] 3. Agree [ ] 4. Strongly agree [ ] |  |
| 23. | I fear being judged by my family or community if I test for HIV. | 1. Strongly disagree [ ] 2. Disagree [ ] 3. Agree [ ] 4. Strongly agree [ ] |  |
| 24. | HIV is often associated with immoral behaviour in my community. | 1. Strongly disagree [ ] 2. Disagree [ ] 3. Agree [ ] 4. Strongly agree [ ] |  |
| 25. | As a man, it is seen as weak to seek HIV testing. | 1. Strongly disagree [ ] 2. Disagree [ ] 3. Agree [ ] 4. Strongly agree [ ] |  |
| 26. | There is too much gossip about people who test for HIV. | 1. Strongly disagree [ ] 2. Disagree [ ] 3. Agree [ ] 4. Strongly agree [ ] |  |
| 27. | My friends think testing for HIV is unnecessary. | 1. Strongly disagree [ ] 2. Disagree [ ] 3. Agree [ ] 4. Strongly agree [ ] |  |
| 28. | I prefer not to talk about sexual health with anyone. | 1. Strongly disagree [ ] 2. Disagree [ ] 3. Agree [ ] 4. Strongly agree [ ] |  |
| 29. | Testing for HIV is not something men in my culture usually do. | 1. Strongly disagree [ ] 2. Disagree [ ] 3. Agree [ ] 4. Strongly agree [ ] |  |

# SECTION E: SOCIOECONOMIC BARRIERS

This section explores how finances, education, access to services, and work-related pressures may limit your ability to test for HIV.

| No. | Question | Response options | Skip |
| --- | --- | --- | --- |
| 30. | I cannot afford transportation to an HIV testing center. | 1. Strongly disagree [ ] 2. Disagree [ ] 3. Agree [ ] 4. Strongly agree [ ] |  |
| 31. | I do not have enough money to pay for health-related costs. | 1. Strongly disagree [ ] 2. Disagree [ ] 3. Agree [ ] 4. Strongly agree [ ] |  |
| 32. | I cannot take time off work to go for an HIV test. | 1. Strongly disagree [ ] 2. Disagree [ ] 3. Agree [ ] 4. Strongly agree [ ] |  |
| 33. | I live far from the nearest testing center. | 1. Strongly disagree [ ] 2. Disagree [ ] 3. Agree [ ] 4. Strongly agree [ ] |  |
| 34. | Health services in my area are of poor quality. | 1. Strongly disagree [ ] 2. Disagree [ ] 3. Agree [ ] 4. Strongly agree [ ] |  |
| 35. | I do not have health insurance to support testing. | 1. Strongly disagree [ ] 2. Disagree [ ] 3. Agree [ ] 4. Strongly agree [ ] |  |
| 36. | My job does not give me time to seek health care. | 1. Strongly disagree [ ] 2. Disagree [ ] 3. Agree [ ] 4. Strongly agree [ ] |  |
| 37. | The cost of testing or related services is too high. | 1. Strongly disagree [ ] 2. Disagree [ ] 3. Agree [ ] 4. Strongly agree [ ] |  |
| 38. | I lack education about the importance of HIV testing. | 1. Strongly disagree [ ] 2. Disagree [ ] 3. Agree [ ] 4. Strongly agree [ ] |  |
| 39. | I have more pressing financial needs than testing for HIV. | 1. Strongly disagree [ ] 2. Disagree [ ] 3. Agree [ ] 4. Strongly agree [ ] |  |

# SECTION F: PSYCHOLOGICAL BARRIERS

This section asks about emotional and mental barriers such as fear, stress, denial, or anxiety that may affect your willingness to test for HIV.

| 40. | I am afraid of testing HIV-positive. | 1. Strongly disagree [ ] 2. Disagree [ ] 3. Agree [ ] 4. Strongly agree [ ] |  |
| --- | --- | --- | --- |
| 41. | I would rather not know my HIV status | 1. Strongly disagree [ ] 2. Disagree [ ] 3. Agree [ ] 4. Strongly agree [ ] |  |
| 42. | I would rather not know my HIV status. | 1. Strongly disagree [ ] 2. Disagree [ ] 3. Agree [ ] 4. Strongly agree [ ] |  |
| 43. | I believe testing for HIV would make me anxious. | 1. Strongly disagree [ ] 2. Disagree [ ] 3. Agree [ ] 4. Strongly agree [ ] |  |
| 44. | If I test positive, I might lose my partner or family. | 1. Strongly disagree [ ] 2. Disagree [ ] 3. Agree [ ] 4. Strongly agree [ ] |  |
| 45. | I do not feel strong enough emotionally to test. | 5. Strongly disagree [ ] 6. Disagree [ ] 7. Agree [ ] 8. Strongly agree [ ] |  |
| 46. | I think I am not at risk for HIV, so I don’t need to test. | 1. Strongly disagree [ ] 2. Disagree [ ] 3. Agree [ ] 4. Strongly agree [ ] |  |
| 47. | I don’t trust health workers to keep my results confidential. | 1. Strongly disagree [ ] 2. Disagree [ ] 3. Agree [ ] 4. Strongly agree [ ] |  |
| 48. | I worry I would be discriminated against if I test positive. | 1. Strongly disagree [ ] 2. Disagree [ ] 3. Agree [ ] 4. Strongly agree [ ] |  |
| 49. | I don’t believe HIV is real or as serious as people say. | 1. Strongly disagree [ ] 2. Disagree [ ] 3. Agree [ ] 4. Strongly agree [ ] |  |

# SECTION G: KNOWLEDGE OF HIV TESTING

This section assesses your understanding of key facts about HIV testing. It aims to evaluate what you know about the purpose, availability, procedures, and implications of HIV testing. Your responses will help identify knowledge gaps and guide strategies to improve HIV testing awareness and uptake.

| 50. | HIV testing is the only way to know a person’s HIV status. | 1. Strongly disagree [ ] 2. Disagree [ ] 3. Agree [ ] 4. Strongly agree [ ] |  |
| --- | --- | --- | --- |
| 51. | HIV can be transmitted during unprotected sex with an infected person. | 1. Strongly disagree [ ] 2. Disagree [ ] 3. Agree [ ] 4. Strongly agree [ ] |  |
| 52. | A person who looks healthy can still have HIV. | 1. Strongly disagree [ ] 2. Disagree [ ] 3. Agree [ ] 4. Strongly agree [ ] |  |
| 53. | HIV testing is available at most health facilities. | 1. Strongly disagree [ ] 2. Disagree [ ] 3. Agree [ ] 4. Strongly agree [ ] |  |
| 54. | Early testing for HIV can lead to better health outcomes. | 1. Strongly disagree [ ] 2. Disagree [ ] 3. Agree [ ] 4. Strongly agree [ ] |  |
| 55. | HIV testing is necessary even if I do not feel sick. | 1. Strongly disagree [ ] 2. Disagree [ ] 3. Agree [ ] 4. Strongly agree [ ] |  |
| 56. | HIV testing is recommended for pregnant women during antenatal care. | 1. Strongly disagree [ ] 2. Disagree [ ] 3. Agree [ ] 4. Strongly agree [ ] |  |
| 57. | HIV testing is free in public health facilities in my area. | 1. Strongly disagree [ ] 2. Disagree [ ] 3. Agree [ ] 4. Strongly agree [ ] |  |
| 58. | HIV testing requires informed consent and confidentiality. | 1. Strongly disagree [ ] 2. Disagree [ ] 3. Agree [ ] 4. Strongly agree [ ] |  |
| 59. | A negative HIV test always means a person does not have HIV | 1. Strongly disagree [ ] 2. Disagree [ ] 3. Agree [ ] 4. Strongly agree [ ] |  |
| 60. | HIV tests can sometimes give a false-negative result if taken too soon after exposure. | 1. Strongly disagree [ ] 2. Disagree [ ] 3. Agree [ ] 4. Strongly agree [ ] |  |
| 61. | HIV testing should be done regularly if one is sexually active. | 1. Strongly disagree [ ] 2. Disagree [ ] 3. Agree [ ] 4. Strongly agree [ ] |  |
| 62. | People who test HIV-positive can live long, healthy lives with treatment. | 1. Strongly disagree [ ] 2. Disagree [ ] 3. Agree [ ] 4. Strongly agree [ ] |  |
| 63. | HIV testing services are also available for couples. | 1. Strongly disagree [ ] 2. Disagree [ ] 3. Agree [ ] 4. Strongly agree [ ] |  |
| 64. | Knowing my HIV status helps protect others and myself. | 1. Strongly disagree [ ] 2. Disagree [ ] 3. Agree [ ] 4. Strongly agree [ ] |  |

# SECTION H: ATTITUDES TOWARD HIV TESTING

This section assesses your evaluations and beliefs regarding HIV testing. The items measure perceived health benefits, emotional reassurance, responsibility, treatment benefits, relationship protection, and perceived disadvantages of HIV testing.

| 65. | Getting tested for HIV will help protect my health. | 1. Strongly disagree [ ] 2. Disagree [ ] 3. Agree [ ] 4. Strongly agree [ ] |  |
| --- | --- | --- | --- |
| 66. | Knowing my HIV status will give me peace of mind. | 1. Strongly disagree [ ] 2. Disagree [ ] 3. Agree [ ] 4. Strongly agree [ ] |  |
| 67. | If I test positive for HIV, I can begin treatment early. | 1. Strongly disagree [ ] 2. Disagree [ ] 3. Agree [ ] 4. Strongly agree [ ] |  |
| 68. | HIV testing will help me protect my sexual partner(s). | 1. Strongly disagree [ ] 2. Disagree [ ] 3. Agree [ ] 4. Strongly agree [ ] |  |
| 69. | Getting tested for HIV is a responsible thing to do. | 1. Strongly disagree [ ] 2. Disagree [ ] 3. Agree [ ] 4. Strongly agree [ ] |  |
| 70. | HIV testing will improve my quality of life. | 1. Strongly disagree [ ] 2. Disagree [ ] 3. Agree [ ] 4. Strongly agree [ ] |  |
| 71. | Knowing my HIV status will help me plan for my future. | 1. Strongly disagree [ ] 2. Disagree [ ] 3. Agree [ ] 4. Strongly agree [ ] |  |
| 72. | Getting tested for HIV is a waste of time. | 1. Strongly disagree [ ] 2. Disagree [ ] 3. Agree [ ] 4. Strongly agree [ ] |  |
| 73. | HIV testing would cause unnecessary stress in my life. | 1. Strongly disagree [ ] 2. Disagree [ ] 3. Agree [ ] 4. Strongly agree [ ] |  |
| 74. | Knowing my HIV status would make me feel safer in my relationships. | 1. Strongly disagree [ ] 2. Disagree [ ] 3. Agree [ ] 4. Strongly agree [ ] |  |

# SECTION I: SUBJECTIVE NORMS

This section assesses perceived social expectations and approval regarding HIV testing from significant others, including partners, family members, peers, religious leaders, healthcare professionals, and the wider community.

| 75. | My sexual partner would like me to get tested for HIV. | 1. Strongly disagree [ ] 2. Disagree [ ] 3. Agree [ ] 4. Strongly agree [ ] |  |
| --- | --- | --- | --- |
| 76. | My family would support me if I decided to get tested for HIV. | 1. Strongly disagree [ ] 2. Disagree [ ] 3. Agree [ ] 4. Strongly agree [ ] |  |
| 77. | My close friends think getting tested for HIV is important. | 1. Strongly disagree [ ] 2. Disagree [ ] 3. Agree [ ] 4. Strongly agree [ ] |  |
| 78. | Religious leaders in my community encourage people to get tested for HIV. | 1. Strongly disagree [ ] 2. Disagree [ ] 3. Agree [ ] 4. Strongly agree [ ] |  |
| 79. | Healthcare professionals encourage men like me to get tested for HIV. | 1. Strongly disagree [ ] 2. Disagree [ ] 3. Agree [ ] 4. Strongly agree [ ] |  |
| 80. | Most men in my community believe HIV testing is important. | 1. Strongly disagree [ ] 2. Disagree [ ] 3. Agree [ ] 4. Strongly agree [ ] |  |
| 81. | People who are important to me would respect me if I got tested for HIV. | 1. Strongly disagree [ ] 2. Disagree [ ] 3. Agree [ ] 4. Strongly agree [ ] |  |
| 82. | People who are important to me would look down on me if I got tested for HIV. | 1. Strongly disagree [ ] 2. Disagree [ ] 3. Agree [ ] 4. Strongly agree [ ] |  |
| 83. | People in my community would think badly of me if I got tested for HIV. | 1. Strongly disagree [ ] 2. Disagree [ ] 3. Agree [ ] 4. Strongly agree [ ] |  |
| 84. | I feel social pressure from people around me to know my HIV status. | 1. Strongly disagree [ ] 2. Disagree [ ] 3. Agree [ ] 4. Strongly agree [ ] |  |

# SECTION J: PERCEIVED BEHAVIOURAL CONTROL (PBC)

This section assesses perceived ability, confidence, autonomy, and access to resources required to undergo HIV testing. It also captures perceived structural and psychological barriers that may influence testing intentions.

| 85. | If I decided to get tested, I am confident that I could do it. | 1. Strongly disagree [ ] 2. Disagree [ ] 3. Agree [ ] 4. Strongly agree [ ] |  |
| --- | --- | --- | --- |
| 86. | Even if I were afraid of the test result, I could still get tested. | 1. Strongly disagree [ ] 2. Disagree [ ] 3. Agree [ ] 4. Strongly agree [ ] |  |
| 87. | I know where I can obtain an HIV test. | 1. Strongly disagree [ ] 2. Disagree [ ] 3. Agree [ ] 4. Strongly agree [ ] |  |
| 88. | HIV testing services are available when I need them. | 1. Strongly disagree [ ] 2. Disagree [ ] 3. Agree [ ] 4. Strongly agree [ ] |  |
| 89. | I can afford any costs associated with getting tested. | 5. Strongly disagree [ ] 6. Disagree [ ] 7. Agree [ ] 8. Strongly agree [ ] |  |
| 90. | I can make enough time to get tested. | 1. Strongly disagree [ ] 2. Disagree [ ] 3. Agree [ ] 4. Strongly agree [ ] |  |
| 91. | I could get tested without other people knowing if I wanted to. | 1. Strongly disagree [ ] 2. Disagree [ ] 3. Agree [ ] 4. Strongly agree [ ] |  |
| 92. | The distance to an HIV testing centre would prevent me from getting tested. | 1. Strongly disagree [ ] 2. Disagree [ ] 3. Agree [ ] 4. Strongly agree [ ] |  |
| 93. | Lack of privacy at the testing facility would prevent me from getting tested. | 1. Strongly disagree [ ] 2. Disagree [ ] 3. Agree [ ] 4. Strongly agree [ ] |  |
| 94. | Whether or not I get tested is entirely my own decision. | 1. Strongly disagree [ ] 2. Disagree [ ] 3. Agree [ ] 4. Strongly agree [ ] |  |

Thank you for completing this questionnaire.

Your response is valuable and will help improve HIV testing strategies for men in Ghana. If you have any concerns or would like more information about this study, feel free to contact the research team at [izyanjr@gmail.com].

We truly appreciate your contribution.
