# Supplementary material for: Determinants of HIV testing intentions among heterosexual men in the Bolgatanga Municipality of Ghana: Evidence from the theory of planned behaviour
Source: PLOS Glob Public Health. 2026 Aug 17;6(8):e0007096. doi: 10.1371/journal.pgph.0007096 (PMC13480663; doi:10.1371/journal.pgph.0007096)
Supplement: Supplementary file 2 [file pgph.0007096.s003.docx]

| Sensitivity analysis comparing standard logistic regression and penalised logistic regression estimates for predictors of strong HIV testing intention | | | | | | | | | | | | |
| --- | --- | --- | --- | --- | --- | --- | --- | --- | --- | --- | --- | --- |
| **Standard Logistic Regression** | | | | |  | **Penalised Logistic Regression** | | | | |  |  |
| Variable | 0dds ratio | P>\|z\| | [95% conf. interval] | |  | Variable | 0dds ratio | P>\|z\| | [95% conf. interval] | |  | Interpretation |
| Attitude towards HIV testing | | |  |  |  | Attitude towards HIV testing | | |  |  |  |  |
| Positive attitude | 1.554458 | 0.024 | 1.213496 | 3.243578 |  | Positive attitude | 1.497223 | 0.026 | 1.237116 | 3.041144 |  | Significant in both models |
| Negative attitude | 1 |  |  |  |  | Negative attitude | 1 |  |  |  |  |  |
| Subjective norms | |  |  |  |  | Subjective norms | |  |  |  |  |  |
| High subjective norms | 2.227278 | 0.022 | 1.595927 | 5.171381 |  | High subjective norms | 2.105411 | 0.017 | 1.137009 | 4.730751 |  | Significant in both models |
| Low subjective norms | 1 |  |  |  |  | Low subjective norms | 1 |  |  |  |  |  |
| Perceived behavioural control (PBC) | | | |  |  | Perceived behavioural control (PBC) | | | |  |  |  |
| High PBC | 2.972531 | 0.017 | 1.762486 | 5.358833 |  | High PBC | 2.800162 | 0.012 | 1.786077 | 5.974727 |  | Significant in both models |
| Low PBC | 1 |  |  |  |  | Low PBC | 1 |  |  |  |  |  |
| Age |  |  |  |  |  | Age |  |  |  |  |  |  |
| 18-27 | 0.459969 | 0.029 | 0.183579 | 0.911129 |  | 18-27 | 2.17479 | 0.018 | 1.360805 | 5.825143 |  | Significant in both models |
| 28-37 | 0.33508 | 0.003 | 0.165144 | 0.695429 |  | 28-37 | 1.299023 | 0.022 | 1.165621 | 3.624107 |  | Significant in both models |
| 38-47 | 0.264066 | 0.121 | 0.187479 | 1.085615 |  | 38-47 | 1.927651 | 0.161 | 0.769577 | 4.828417 |  | Not statistically significant in both models |
| 48+ | 1 |  |  |  |  | 48+ | 1 |  |  |  |  |  |
| Marital status | |  |  |  |  | Marital status | |  |  |  |  |  |
| Unmarried | 0.860262 | 0.229 | 0.220057 | 3.362998 |  | Unmarried | 0.812699 | 0.751 | 0.226255 | 2.91919 |  | Not statistically significant in both models |
| Married | 1.421968 | 0.152 | 0.243809 | 3.118494 |  | Married | 0.828355 | 0.757 | 0.251871 | 2.724297 |  | Not statistically significant in both models |
| Divorced | 1 |  |  |  |  | Divorced | 1 |  |  |  |  |  |
| Educational attainment | | |  |  |  | Educational attainment | | |  |  |  |  |
| No formal education | 0.405235 | 0.215 | 0.245013 | 3.594373 |  | No formal education | 0.569012 | 0.547 | 0.090898 | 3.561941 |  | Not statistically significant in both models |
| Primary School | 1.379951 | 0.121 | 0.235612 | 3.082196 |  | Primary School | 1.516148 | 0.611 | 0.305545 | 7.523286 |  | Not statistically significant in both models |
| Junior High School | 0.375493 | 0.115 | 0.111104 | 1.269034 |  | Junior High School | 0.401481 | 0.123 | 0.125864 | 1.28064 |  | Not statistically significant in both models |
| Senior High School | 0.552311 | 0.182 | 0.231004 | 1.320529 |  | Senior High School | 0.567712 | 0.185 | 0.245908 | 1.310643 |  | Not statistically significant in both models |
| Tertiary | 0.882092 | 0.164 | 0.394536 | 1.972157 |  | Tertiary | 0.869021 | 0.722 | 0.40135 | 1.881644 |  | Not statistically significant in both models |
| Vocational School | 1 |  |  |  |  | Vocational School | 1 |  |  |  |  |  |
| Monthly income | |  |  |  |  | Monthly income | |  |  |  |  |  |
| Ghȼ 100 [$9.7] and Ghȼ 500 [$45.35] | 0.765549 | 0.227 | 0.170809 | 3.431115 |  | Ghȼ 100 [$9.7] and Ghȼ 500 [$45.35] | 0.746802 | 0.69 | 0.178195 | 3.129787 |  | Not statistically significant in both models |
| Ghȼ 600 [$54.4] and Ghȼ 1000 [$90.7] | 1.587412 | 0.199 | 0.41545 | 3.065407 |  | Ghȼ 600 [$54.4] and Ghȼ 1000 [$90.7] | 1.499595 | 0.534 | 0.417576 | 5.385338 |  | Not statistically significant in both models |
| Ghȼ 1100 [$99.8] and Ghȼ 2000 [$181.5] | 2.065556 | 0.216 | 0.581505 | 5.337028 |  | Ghȼ 1100 [$99.8] and Ghȼ 2000 [$181.5] | 1.899144 | 0.298 | 0.568175 | 6.347949 |  | Not statistically significant in both models |
| Ghȼ 2100 [$190.5] and Ghȼ 3000 [$272.2] | 1.900838 | 0.218 | 0.681907 | 3.209213 |  | Ghȼ 2100 [$190.5] and Ghȼ 3000 [$272.2] | 1.756635 | 0.326 | 0.570357 | 5.410242 |  | Not statistically significant in both models |
| Ghȼ 3100 [$280.8] and Ghȼ 4000 [$362.3] | 1 |  |  |  |  | Ghȼ 3100 [$280.8] and Ghȼ 4000 [$362.3] | 1 |  |  |  |  |  |
| Media exposure [Listening to the radio, watching television, and internet usage] | | | | | | Media exposure [Listening to the radio, watching television, and internet usage] | | | | | | |
| Almost every day | 2.115859 | 0.011 | 1.527967 | 3.665955 |  | Almost every day | 2.165182 | 0.027 | 1.574767 | 5.36209 |  | Significant in both models |
| At least once a week | 2.784688 | 0.016 | 1.873613 | 4.564442 |  | At least once a week | 2.511963 | 0.033 | 1.570373 | 4.326581 |  | Significant in both models |
| Less than once a week | 1 |  |  |  |  | Less than once a week | 1 |  |  |  |  |  |
| _cons | 0.044436 | 0.002 | 0.006172 | 0.319906 |  | _cons | 0.06199 | 0.003 | 0.00962 | 0.399468 |  |  |
