# Supplementary material for: Determinants of HIV testing intentions among heterosexual men in the Bolgatanga Municipality of Ghana: Evidence from the theory of planned behaviour
Source: PLOS Glob Public Health. 2026 Aug 17;6(8):e0007096. doi: 10.1371/journal.pgph.0007096 (PMC13480663; doi:10.1371/journal.pgph.0007096)
Supplement: Supplementary file 3 [file pgph.0007096.s004.docx]

| Component | Initial Eigenvalue | % of Variance Explained | Cumulative % |
| --- | --- | --- | --- |
| Factor 1 | 10.88 | 32 | 32 |
| Factor 2 | 3.95 | 11.62 | 43.62 |
| Factor 3 | 2.85 | 8.38 | 52 |
| Factor 4 | 1.95 | 5.74 | 57.74 |
